# Resilient Cooperative Control of Connected and Automated Mixed Traffic: From Effective Controllability to Network Recovery

**DOI:** 10.3390/s26196294

**Published:** 2026-10-04

**Authors:** Haoda Fang, Yilin Wang, Zhangcheng Yin, Wenting Yang, Zhiyi Tang, Yating Hong, Mengyu Sun, Yaochen Lin, Miergule Tuohetibulati, Yu Xiang

**Affiliations:** 1 Xinjiang Key Laboratory of Green Construction and Smart Traffic Control of Transportation Infrastructure, Xinjiang University, Urumqi 830017, China; 107552401776@stu.xju.edu.cn; 2 School of Traffic and Transportation Engineering, Xinjiang University, Urumqi 830017, China; wangyilin3098@stu.xju.edu.cn (Y.W.); 20232008204@stu.xju.edu.cn (Z.Y.); 20252802001@stu.xju.edu.cn (W.Y.); tangzhiyi@stu.xju.edu.cn (Z.T.); 15256658291@163.com (Y.H.); 20252802006@stu.xju.edu.cn (M.S.); mier9137@163.com (M.T.); 3 College of Civil Engineering and Architecture, Xinjiang University, Urumqi 830017, China; alin_12096878@163.com

**Keywords:** connected and automated vehicles, mixed traffic, cooperative control, traffic resilience, effective controllability, signal–vehicle coordination, infrastructure sensing, communication-aware control

## Abstract

**Highlights:**

**What are the main findings?**

- Effective CAV controllability depends on spatial availability, HDV response, platoon organization, and information and computation quality—not on penetration alone.
- Cross-scale resilience requires coupling vehicle/platoon actuation with signal, routing, and regional traffic control while evaluating service retention, cumulative loss, and recovery time.

**What are the implications of the main findings?**

- V2X quality should be treated as a control constraint that triggers graded cooperation, safe fallback, and re-synchronization rather than as an ideal communication assumption.
- Deployment evidence should progress from simulation to real-data, SIL/HIL, and field validation under reproducible physical and communication disturbances.

**Abstract:**

Connected and automated vehicles (CAVs) are emerging as mobile sensing and actuation resources in mixed traffic, but their contribution to urban road-network resilience depends on more than nominal penetration. This structured critical review examines how local CAV sensing and control can be translated into effective controllability and measurable recovery across vehicle, intersection, corridor, and network scales. An auditable pre-submission update retrieved 1601 raw records from the Web of Science Core Collection, ScienceDirect, and IEEE Xplore and yielded 1476 unique auditable candidates after deduplication. This update identified 66 incremental relevant records, which were combined with the earlier 202-study evidence pool to form a 268-record corpus for the final synthesis. The final corpus was organized into core, bridging, and background/methodological evidence and synthesized using an eight-dimensional evidence framework. The synthesis shows that effective controllability depends on CAV spatial availability, platoon organization, HDV response, communication quality, and computation latency. Signal–vehicle coordination can jointly reshape right-of-way and arrival processes, yet most studies still emphasize nominal delay, energy, throughput, or safety rather than disruption-stage service retention and recovery. We therefore synthesize a cross-scale control architecture and a communication-aware control-quality contract linking traffic and V2X states, cooperation mode, safe fallback, and re-synchronization. Service retention, cumulative performance loss, and recovery time are recommended as resilience objectives, while the E0–E4 maturity ladder highlights the remaining gap between simulation performance and deployment-ready evidence.

## 1. Introduction

Urban road networks are critical infrastructure whose service can deteriorate abruptly under crashes, work zones, extreme demand, natural hazards, infrastructure failures, and cyber–physical disruptions. Resilience therefore concerns more than resistance to the initial performance drop: it also concerns how much essential mobility is retained. It also concerns how long the disturbance persists and how quickly acceptable operation is restored afterward [1,2,3,4,5,6]. For control research, this shifts the central question from ‘How well does the controller perform in nominal traffic?’ to ‘How does the controller shape the complete disturbance-recovery trajectory?’

CAVs create a new control resource within this resilience problem. Unlike HDVs, whose decisions depend primarily on local perception and human response, CAVs can combine onboard sensing with V2V/V2I/V2X information. They can alter traffic states through speed regulation, spacing control, trajectory planning, cooperative merging, and platooning [7,8,9,10,11,12,13]. These capabilities make CAVs mobile sensors and actuators, but their dependence on sensing and communication also creates additional failure, delay, and attack pathways [14,15].

Resilience-oriented mixed-traffic control thus requires two coupled functions. Infrastructure, signal, routing, and perimeter controllers allocate scarce right-of-way and residual network capacity, whereas controllable vehicles shape arrivals, shock waves, headways, and local conflicts inside the traffic stream. Neither layer is sufficient in isolation. A deployable architecture must coordinate vehicle/platoon, intersection/corridor, and regional-network decisions while explicitly accounting for information quality and safe fallback. In addition to conventional roadside and vehicle sensing, material-integrated structural sensing can provide an upstream estimate of physical infrastructure condition when deterioration, cracking, or load-induced damage changes usable capacity before traffic measurements alone reveal the full consequence.

Recent reviews provide strong coverage of mixed-traffic capacity, stability, safety, and environmental impacts [16]; urban mixed-traffic signal, trajectory, routing, and lane control [17]; safety and efficiency [18]; positive and negative externalities of mixed autonomy [19]; optimization-based traffic and fleet management [20]; mixed-traffic signal control [21]; reinforcement-learning-based signal/vehicle control [22]; and multi-intersection cooperative-control platforms [23]. A complementary 2026 survey in IEEE Communications Surveys & Tutorials organizes V2X-enabled cooperative safety intelligence through a closed sensing–perception–decision framework. It explicitly treats latency, synchronization, bandwidth, packet/feature loss, reliability, trust, and practical deployment conditions as coupled constraints [24]. These surveys establish important foundations, but their organizing axes are mainly technology layer, algorithm family, nominal performance, safety intelligence, or implementation platform. Control synthesis is missing: the mechanism by which locally available CAV authority propagates through traffic and infrastructure layers to produce measurable service retention and network recovery under disturbance.

The closest recent synthesis is the 2026 PRISMA-ScR review by Ortega et al. [25], which mapped 106 peer-reviewed studies to robustness, redundancy, resourcefulness, and rapidity and found much thinner evidence for recovery and field validation than for simulation-based efficiency. The present review is deliberately narrower and more control-oriented: Under what traffic compositions, information conditions, disturbance types, and control architectures can CAV sensing and actuation be converted into measurable resilience at the urban road-network level?

**Figure 1:**
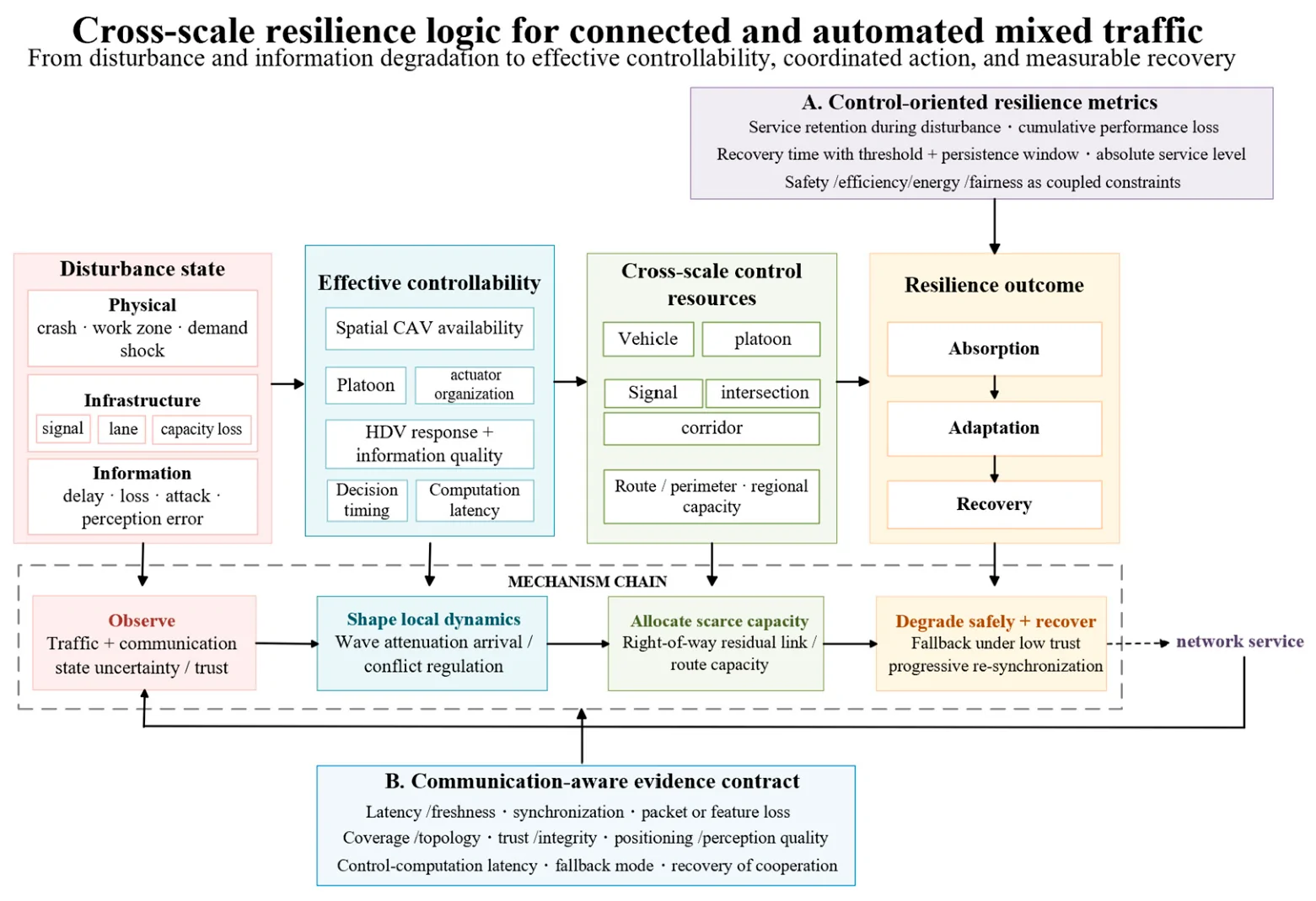
Unified disturbance-effective–controllability–cross-scale-control–resilience framework and review roadmap. Colors distinguish the major conceptual modules, while arrows indicate the direction of information/control influence across the disturbance–control–resilience chain.

Figure 1 integrates the review’s causal and organizational logic. Disturbances may originate in the physical, infrastructure, or information layer; nominal CAV availability is filtered through spatial location, platoon organization, HDV response, and information quality to determine effective controllability; local actions then reshape waves, arrivals, conflicts, and queues before regional signals, routing, perimeter control, and residual-capacity allocation can influence network service. Resilience is evaluated across absorption, adaptation, and recovery rather than by nominal efficiency alone. The same figure maps this mechanism to RQ1–RQ4, so the review follows one explanatory chain from disturbance state to controllability, coordinated action, measurable recovery, and engineering evidence.

This review makes four contributions. First, it develops an eight-dimensional evidence framework that separates nominal CAV penetration from effective controllability and identifies spatial availability, HDV response, platoon organization, information quality, and computation timing as mediating conditions. Second, it maps vehicle/platoon control, signal control, signal–vehicle coordination, routing, and perimeter management to service retention, cumulative loss, and recovery time, thereby separating disruption-stage resilience from normal-operation efficiency. Third, it introduces a communication-aware control-quality contract that treats freshness, synchronization, reliability, bandwidth, coverage, trust, perception quality, and computation latency as variables that determine whether full cooperation, degraded cooperation, safe fallback, or re-synchronization is feasible. Fourth, it combines mechanism-based synthesis with descriptive bibliometrics, an author-defined E0–E4 engineering-evidence ladder, and a staged validation/benchmark architecture to make deployment gaps and reporting requirements explicit.

Four research questions organize the review. RQ1 asks how penetration, spatial distribution, platoon organization, and information quality shape mixed-traffic stability, capacity, and effective controllability under disturbance. RQ2 asks how vehicle/platoon, intersection, corridor, and network control influence wave propagation, queue spillback, residual-capacity use, and recovery. RQ3 asks how online objectives can be aligned with service retention, cumulative loss, recovery time, safety, efficiency, and fairness. RQ4 asks where the main deployment boundaries lie under low penetration, degraded communication/perception, limited computation, and simulation-to-real transfer.

The article is organized to mirror these questions. Section 2 establishes the auditable corpus and evidence architecture; Section 3 defines resilience quantities and disturbance mechanisms; Section 4, Section 5, Section 6 and Section 7 trace the mechanism from mixed-flow controllability to signal–vehicle and network-level control; Section 8 treats V2X, material-integrated infrastructure sensing, computing, benchmarks, and engineering validation as cross-cutting constraints; and Section 9 consolidates evidence strength, deployment boundaries, and a testable research agenda.

Relative to the recent reviews summarized in Table 1, the intended contribution of the present study is not broader coverage of every individual CAV-control topic. Rather, it explicitly links five questions that are usually treated separately: the spatial availability of controllable CAVs, the information conditions under which those vehicles remain observable and actionable, the coupling of vehicle actions with signal/routing/regional control, the measurement of disturbance-stage service retention and recovery, and the engineering-validation boundary supporting each claim. Table 1 therefore compares organizing scope rather than ranking the methodological quality of previous reviews.

## 2. Review Methodology

### 2.1. Research Scope and Evidence Tiers

This study is a structured critical review rather than a statistical meta-analysis. The evidence base is too heterogeneous in network topology, demand, CAV penetration, HDV behavior, communication assumptions, disturbance design, baseline controller, and outcome definition for pooled percentage improvements to be scientifically defensible. Reproducibility is pursued instead through an auditable search update, explicit relevance screening, evidence-tier rules, multidimensional coding, and transparent interpretation boundaries.

The scope is restricted to urban roads and road networks involving CAV-HDV mixed traffic, traffic-signal/vehicle coordination, network-level traffic management, and directly relevant resilience, communication-degradation, cybersecurity, or engineering-validation mechanisms. Evidence is separated into three tiers. Core evidence directly investigates CAV/HDV mixed-traffic control or resilience on urban roads. Bridging evidence supplies transferable mechanisms for disturbance propagation, network resilience, communication failure, cyber-resilience, or validation without directly proving a CAV network effect. Retain background evidence only when established algorithms, traffic-control systems, or validation concepts are required for interpretation.

### 2.2. Data Sources and Search Strategy

The review used a structured, topic-oriented search and evidence-synthesis strategy. The historical search framework covered the Web of Science Core Collection, Scopus, IEEE Xplore, and TRID; ScienceDirect and publisher pages were also used for complementary retrieval and bibliographic/full-text verification. Because complete database-by-database logs from the earliest Scopus and TRID searches were not retained, the review does not reconstruct artificial retrospective counts. Instead, the fully auditable pre-submission update is reported separately and used to anchor the final evidence pool.

On 17 August 2026, the retained update comprised eight Web of Science Core Collection export batches (807 records), seven ScienceDirect batches (570 records), and four targeted IEEE Xplore batches (224 records), yielding 1601 raw records. DOI-first deduplication supplemented by normalized-title matching resulted in 1476 unique auditable candidates. Relevance reassessment of the update identified 66 incremental records that contributed additional control mechanisms, information conditions, validation evidence, or boundary cases beyond the earlier 202-study evidence pool. The earlier pool and the 66 incremental records were then combined to form the final 268-record evidence corpus, comprising 170 core, 89 bridging, and 9 background/methodological studies. Source-specific overlap and batch-level accounting are not used as separate evidence streams and therefore are omitted here for clarity.

Titles, abstracts, keywords, and, where needed, full text were assessed against RQ1–RQ4 and against the incremental mechanism or validation value contributed by each record. We did not mechanically enumerate highly homogeneous studies with repeated assumptions and outcomes in the main text. We prioritized representative evidence that introduced a distinct control scale, disturbance mechanism, information condition, engineering-validation level, failure mode, or boundary condition.

The conceptual search framework used four term groups: A, traffic environment (connected and automated vehicle*, connected autonomous vehicle*, CAV, connected vehicle*, mixed traffic, mixed autonomy); B, control (traffic signal control, cooperative control, trajectory control, eco-driving, platoon*, multi-agent reinforcement learning, MARL, model predictive control); C, resilience/disturbance (resilien*, disrupt*, incident*, capacity reduction, recovery, network failure, communication delay, packet loss, cyberattack*); and D, road context (urban road*, road network*, intersection*, corridor, traffic network). The core logic was (A AND B AND D) OR (A AND C AND D), while (B AND C AND D) was used only for bridging evidence. Platform-specific field limits were handled with shorter equivalent combinations, and forward/backward citation tracking reduced omissions caused by inconsistent terminology.

The exact earliest Scopus and TRID query histories are not available and are therefore not retrospectively reconstructed. For the retained 2026 update, the search logic, databases, retrieval date, term groups, and exported batches reported above constitute the auditable search record. Because platform-specific query syntax and field limits differed among Web of Science, ScienceDirect, and IEEE Xplore, the manuscript reports the common conceptual logic and retained export provenance rather than presenting reconstructed database strings that cannot be independently verified. This limitation is also stated in Section 9.7.

The relationship between the historical evidence pool, the 2026 auditable search update, and the final synthesis corpus is summarized in Table 2.

### 2.3. Inclusion, Exclusion, and Evidence Coding

We retained studies that addressed urban-road, intersection, corridor, or network operations; CAV-HDV mixed traffic or a directly transferable resilience mechanism; signal, trajectory, platoon, lane, route, perimeter, or network control; disturbance propagation or recovery; or communication/perception degradation relevant to those control problems. We excluded pure single-vehicle perception/planning without traffic-operation implications, duplicate or non-auditable material, and cross-modal studies without a transferable mechanism from the core tier. The initial relevance screening and evidence extraction were conducted by the first author using the predefined scope and inclusion/exclusion criteria in Table 3. The final 268-record evidence corpus and all borderline evidence-tier assignments were subsequently checked by the corresponding author for consistency with the predefined scope. The review was not designed as a dual-independent-screening systematic review, and formal inter-reviewer agreement statistics were therefore not calculated. This limitation is stated explicitly to avoid implying a level of screening replication that was not part of the original review design.

Eight evidence dimensions were used to structure the cross-study synthesis: traffic environment; information structure; control object; control method; disturbance type; performance/resilience objective; spatial validation scale; and engineering-validation maturity. These dimensions were used as an interpretive framework rather than as a mutually exclusive numerical scoring system. Individual studies could contribute evidence to more than one dimension or scale.

Within the eighth dimension, E0–E4 is introduced as an author-defined qualitative maturity ladder for interpreting the type of validation supporting a claim: E0 represents analytical or simulation evidence; E1 represents evidence involving real-data calibration or replay; E2 denotes software-in-the-loop validation; E3 denotes hardware-in-the-loop or physical-testbed validation; and E4 denotes a controlled pilot, field experiment, or field operational test. The ladder is not used as a numerical study-quality score, and the manuscript does not imply that an E4 study necessarily produces a larger or more reliable effect size than an E0 study.

The coding fields in Table 4 should therefore be interpreted as a structured extraction and interpretation dictionary, not as an exhaustive mutually exclusive taxonomy or a numerical scoring system. When a study addressed multiple control objects, disturbances, objectives, or scales, all relevant dimensions were retained in the narrative synthesis rather than forcing the study into a single category.

**Figure 2:**
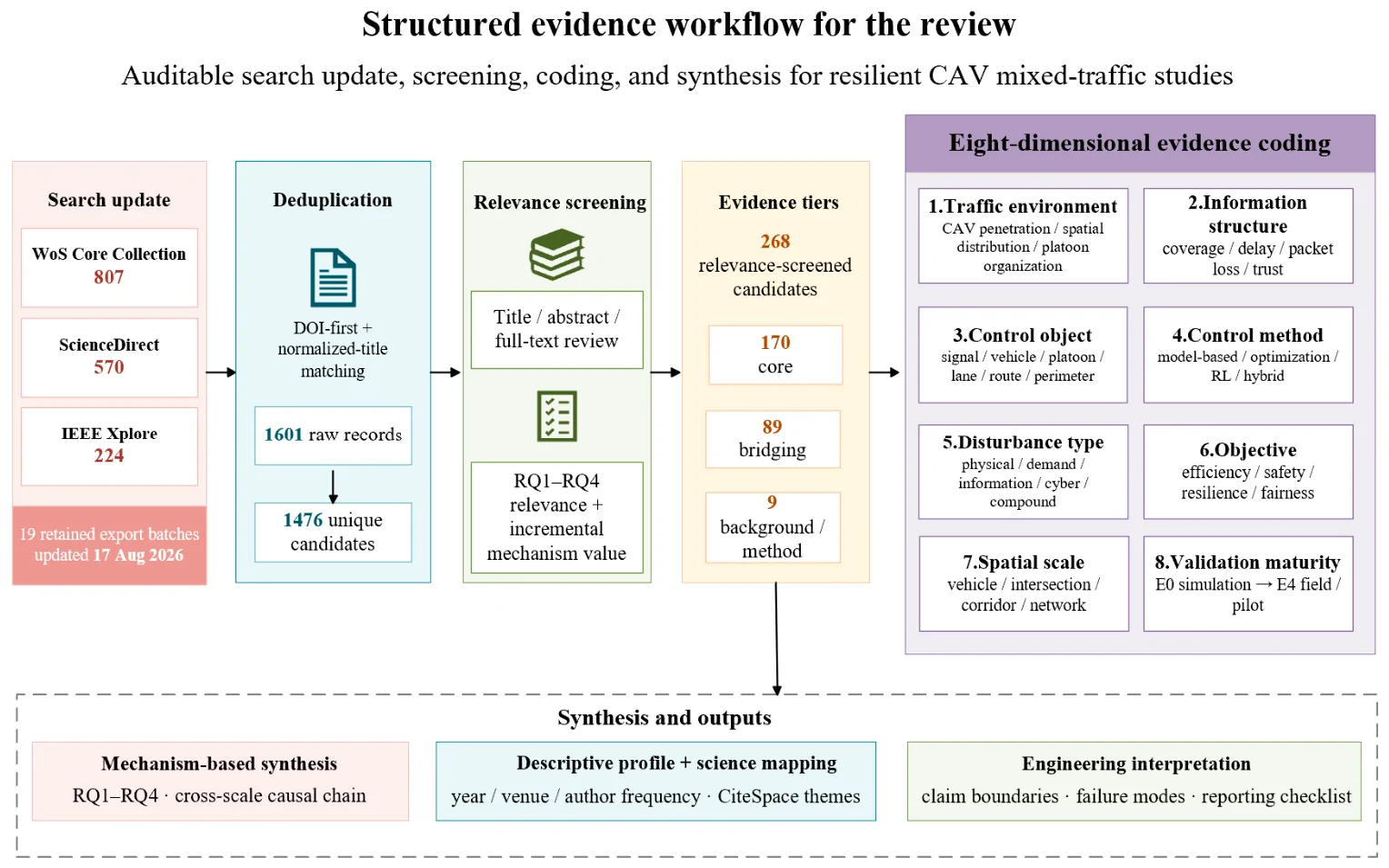
Structured search, screening, evidence-coding, and synthesis workflow.

Figure 2 summarizes the structured search, screening, evidence-coding, and synthesis workflow.

### 2.4. Evidence Synthesis and Quality Interpretation

Algorithms are not ranked by the relative percentage improvement reported in individual papers. Instead, each result is interpreted together with its operating scenario, information availability, action space, safety/feasibility constraints, baseline controller, disturbance design, and validation maturity. Quantitative improvements are treated as scenario-specific evidence unless demand, penetration, baseline information access, disturbance intensity, and evaluation conditions are sufficiently transparent for comparison.

Representative evidence was selected using three principles: direct relevance to an RQ, incremental mechanism or spatial scale, and a clearly stated validation boundary. Priority was given to studies that test low-penetration or degraded-information conditions, span multiple control layers, include disturbances or recovery, provide higher-maturity validation, or reveal failure modes. Evidence quality was assessed based on parameter transparency, baseline fairness, statistical reporting, realism of safety and disturbance assumptions, computation reporting, and independent-network, real-data, SIL, HIL, or field validation.

The review does not claim exhaustive coverage and is not presented as a registered PRISMA systematic review. The retained WoS, ScienceDirect, and IEEE files make the 2026 update auditable, whereas early Scopus/TRID screening counts cannot be reconstructed completely. This limitation is reported explicitly rather than converted into a flow diagram with false precision.

### 2.5. Descriptive Bibliometric Profile and Exploratory Science Mapping

**Figure 3:**
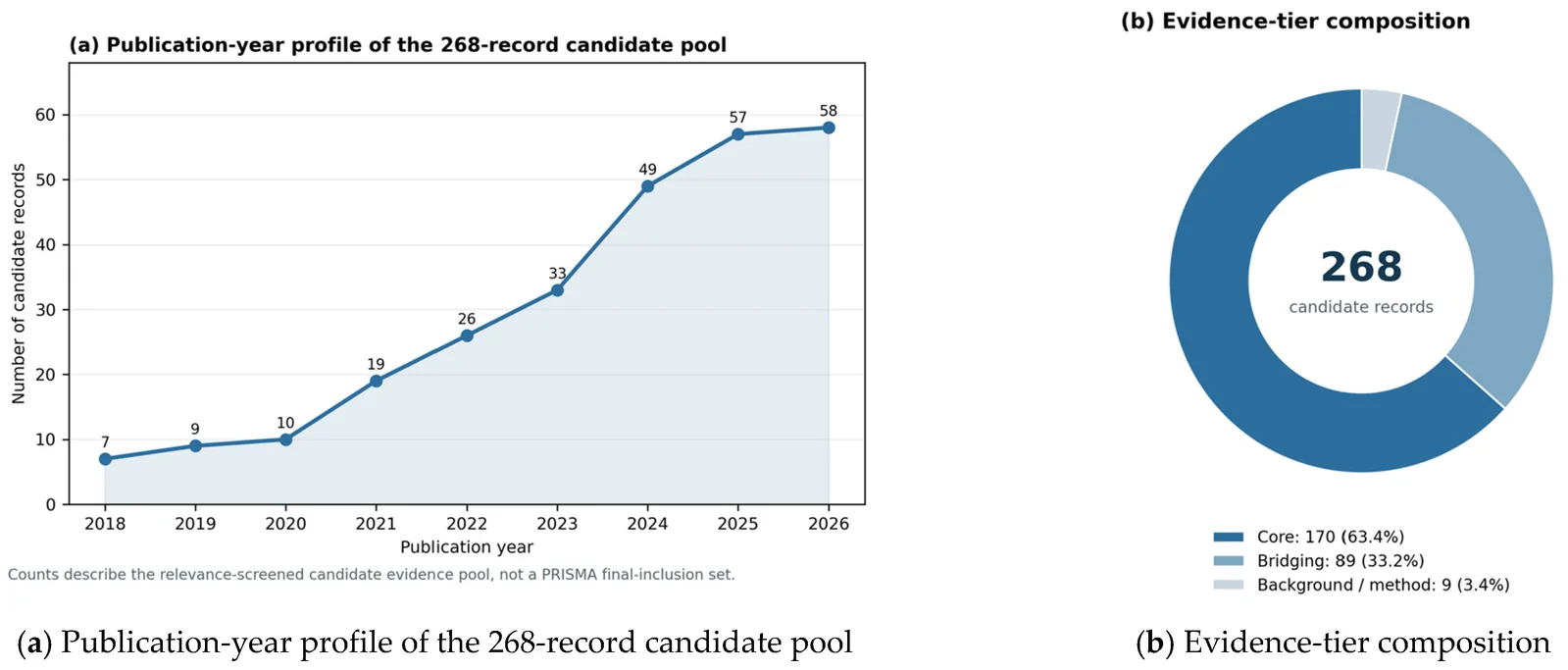
Descriptive profile of the 268-record candidate evidence pool: publication-year distribution (2026 partial through 17 August) and evidence-tier composition.

**Figure 4:**
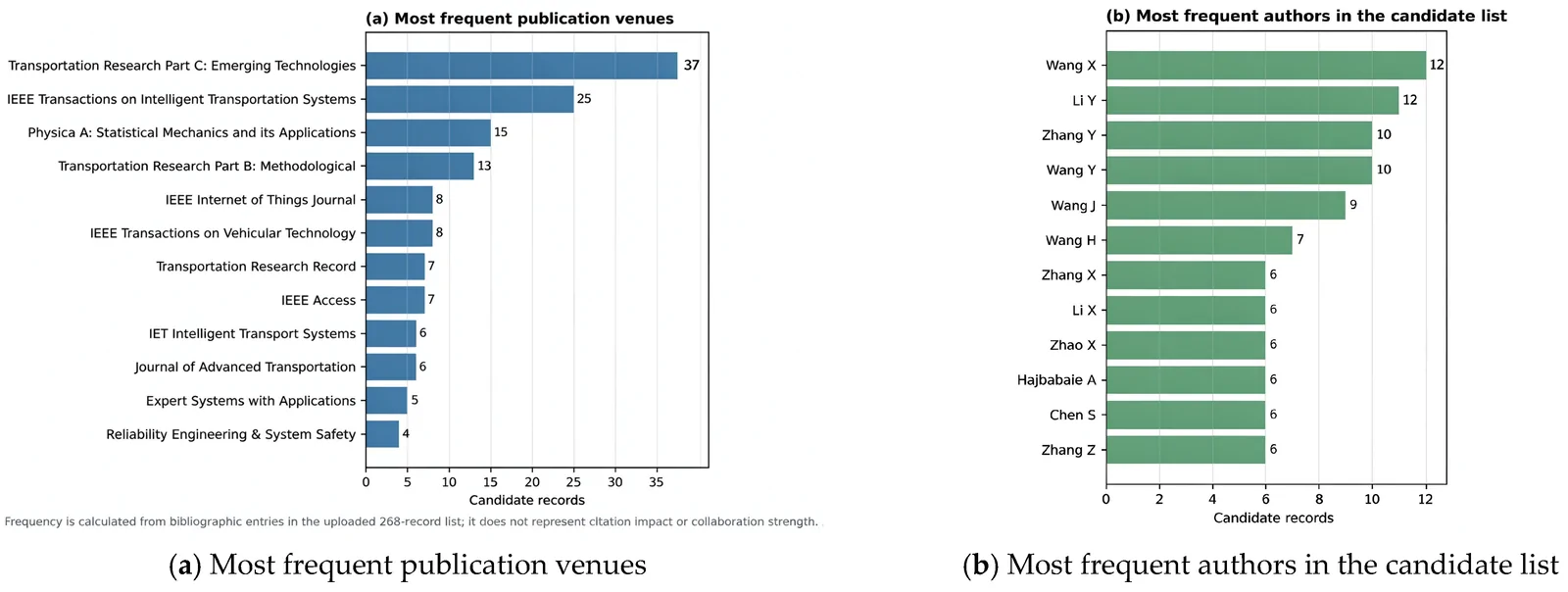
Most frequent publication venues and authors represented in the 268-record candidate list.

A descriptive profile of the 268-record relevance-screened candidate pool was added to complement the mechanism-based synthesis. The pool is strongly recent: annual counts increase from 7 records in 2018 to 58 in 2026 (partial year through 17 August), and 164 records (61.2%) were published during 2024–2026. Core evidence accounts for 170 records (63.4%), bridging evidence for 89 (33.2%), and background/methodological evidence for 9 (3.4%). Transportation Research Part C: Emerging Technologies (37 records) and IEEE Transactions on Intelligent Transportation Systems (25) are the most frequent venues in the candidate list, followed by Physica A (15) and Transportation Research Part B (13). Author-frequency and venue-frequency plots are descriptive only: they do not represent citation impact, institutional influence, or collaboration strength because the uploaded candidate list does not contain complete affiliation and citation metadata. The publication-year and evidence-tier profiles are shown in Figure 3, while the venue and author frequency profiles are presented in Figure 4.

For thematic science mapping, we generated keyword co-occurrence maps in CiteSpace 6.4.R2 using the retained Web of Science records available for visualization [26,27]. The analysis covered 2018–2026 with one-year time slices and g-index selection (k = 25; LRF = 3.0; L/N = 10; LBY = 5; e = 1.0). The unpruned network contained 235 nodes and 1289 links (density = 0.0469), with modularity Q = 0.4207 and weighted mean silhouette S = 0.7468. Pathfinder pruning retained 235 nodes and 584 links (density = 0.0212), with Q = 0.669 and S = 0.856. These values are reported only as descriptive properties of the CiteSpace-generated network. Because no degree-preserving null-model significance test was performed, the modularity and silhouette values are not interpreted here as statistical evidence that the observed thematic structure differs from that expected by chance. The science-mapping analysis is therefore used only as exploratory visualization supporting the narrative synthesis. A complementary keyword-burst analysis over the 2018–2026 window was used to characterize when specific topics experienced concentrated increases in attention.

**Figure 5:**
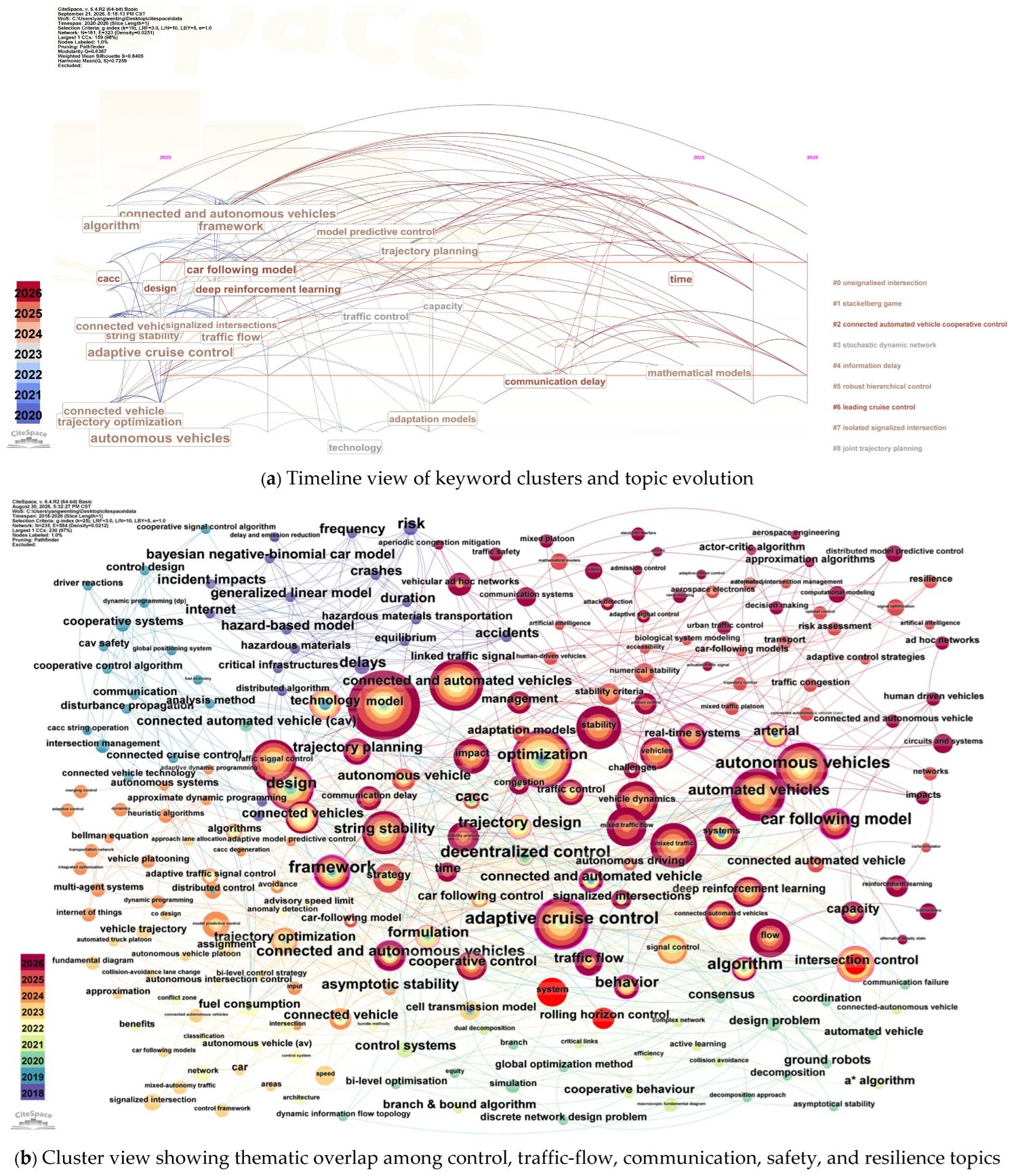
CiteSpace keyword co-occurrence maps for connected and automated mixed-traffic control and resilience research (2018–2026).

Figure 5 provides complementary timeline and cluster views. The timeline indicates a progression from car-following models, adaptive cruise control, decentralized control, and intersection management toward trajectory design, cooperative control, mixed platoons, traffic-signal coordination, communication systems, and explicit road-network resilience. The cluster view shows that control design, traffic flow, communication, safety, HDV behavior, and network management are strongly interwoven rather than isolated themes. This overlap supports the cross-scale organizing logic used in the review. At the same time, the persistence of evaluation and real-time-system terms near the thematic frontier reinforces the distinction between algorithmic novelty and engineering validation.

**Figure 6:**
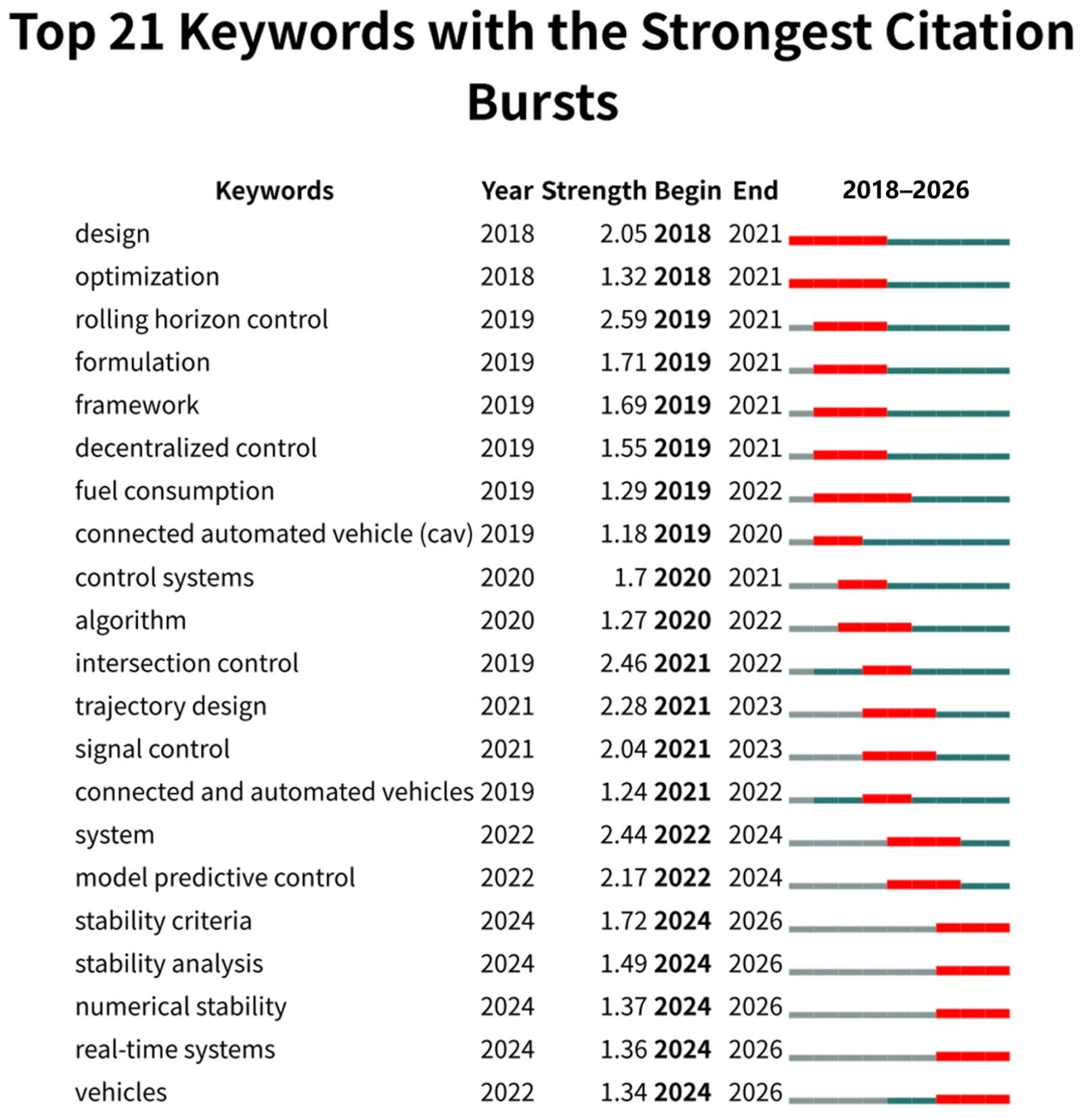
CiteSpace keyword burst detection for 21 highlighted terms over the 2018–2026 analysis window (red segments denote burst periods).

Figure 6 adds a temporal burst perspective to the co-occurrence structure in Figure 5. The strongest early bursts are associated with design and optimization (2018–2021), while rolling-horizon control shows the largest burst strength in the chart (2.59 during 2019–2021). The middle period shifts toward intersection control (2021–2022), trajectory design and signal control (2021–2023), and system-level and model-predictive-control terms (2022–2024). The most recent 2024–2026 bursts concentrate on stability criteria, stability analysis, numerical stability, real-time systems, and vehicles. Read together with Figure 5, this pattern suggests a transition from formulation- and optimization-centered work to stability-conscious, implementation-oriented control. Burst intensity reflects short-term changes in keyword attention rather than effect size, methodological quality, or causal importance, so it is treated here as descriptive triangulation rather than independent engineering evidence.

The main bibliometric themes and their roles in the cross-scale synthesis are summarized in Table 5.

Accordingly, the bibliometric outputs are used as triangulation rather than as independent causal evidence. Descriptive counts, co-occurrence maps, and keyword-burst patterns show where research activity is concentrated and how vocabulary and temporal attention have evolved; all engineering claims in subsequent sections remain grounded in primary-study mechanisms, assumptions, disturbance conditions, and validation boundaries.

### 2.6. Transparency and Reproducibility Notes

Search-accounting statements in this manuscript follow the retained 2026 export files and the 268-record candidate list. Descriptive bibliometric counts were computed only from bibliographic fields present in that list; country, institution, citation-impact, bibliographic-coupling, and co-authorship metrics were deliberately not reconstructed because the uploaded evidence list does not contain the complete metadata required for defensible analysis. The 12 self-sensing UHPC papers discussed in Section 8.2 are an explicitly labeled targeted bridge set and are excluded from all search-accounting and science-mapping statistics. Generative AI was used only to assist English-language refinement and journal-oriented restructuring of author-generated text; it was not used to create search counts, screening outcomes, bibliographic records, or evidence-tier assignments. The authors remain responsible for final scientific verification and journal-specific disclosure.

## 3. Urban Road-Network Resilience: Concepts, Metrics, and Disturbance Mechanisms

### 3.1. Definition and Engineering Interpretation

For this review, resilience is interpreted as an urban road network’s ability to preserve essential mobility under disturbance and regain an acceptable operating state afterward. The disturbance-response trajectory is separated into three functions: absorption, which limits the initial performance loss; adaptation, which retains service while the disturbance persists through control, rerouting, or resource reconfiguration; and recovery, which removes residual congestion and restores acceptable operation.

Resilience differs from robustness because it depends on both the magnitude and the duration of service loss. A controller may keep performance relatively high during a disruption but recover slowly, while another may experience a deeper initial drop yet restore service rapidly. Normalized resilience scores can also be misleading when the pre-disturbance baseline is weak. Reporting should therefore pair relative loss with absolute service levels and should state the baseline, disturbance window, and recovery criterion explicitly [28,29,30].

CAV deployment makes resilience explicitly cyber–physical. Conventional assessments focus on topology and traffic-flow performance; connected traffic additionally depends on communication reachability, delay, packet delivery, perception quality, information integrity, and recovery from information-layer failures. Physical capacity loss can interact with degraded information, and nominal CAV penetration can overstate the fraction of vehicles that are actually observable and controllable. The relevant quantity is therefore functional service supported by jointly available road, vehicle, sensing, communication, and control resources.

### 3.2. Control-Oriented Resilience Quantification

Urban road-network resilience is measured using five complementary families of methods: topological connectivity, traffic-flow performance, resilience-triangle/performance-curve measures, macroscopic fundamental diagrams (MFDs), and cyber–physical coupled metrics. Topological measures efficiently identify critical nodes and redundancy but do not represent queue formation or dissipation directly [31]. Traffic-flow measures are service-oriented but require a clear baseline and disturbance interval. MFDs provide low-dimensional network states useful for regional assessment and perimeter control [32,33]. Cyber–physical measures are essential when communication or perception quality changes the amount of functional control authority.

No single metric family is sufficient for control evaluation. At minimum, resilience studies should report: (i) the pre-disturbance baseline; (ii) the absolute service level retained during disruption; (iii) cumulative loss over the disturbance-recovery horizon; and (iv) recovery time using an explicit threshold and persistence rule. These should be paired with operational indicators such as speed, travel time, throughput, queue length, completion rate, safety risk, energy use, or fairness [34,35].

Let *P*(*t*) denote a performance indicator and *P*_0_ its pre-disturbance baseline. For benefit-type metrics (e.g., throughput), normalized service is defined as

(1) $$S(t)=\frac{P(t)}{P_{0}}$$

For cost-type metrics (e.g., delay), normalized service is defined as

(2) $$S(t)=\frac{P_{0}}{P(t)}$$

Equations (1) and (2) are intended only for strictly positive ratio-scale indicators for which *P*_0_ > 0 and, for the cost-type definition, *P_t_* > 0 throughout the evaluated interval. They should not be applied to zero-baseline or sign-changing quantities. In such cases, the absolute performance measure should be reported directly, or an affine normalization should be defined using two physically meaningful fixed reference levels rather than division by *P*_0_. Thus, the normalization used in this review is a reporting convention for compatible traffic-service indicators, not a universal transformation for arbitrary performance variables.

A unified loss function is defined as

*L*(*t*) = max{0, 1 − *S*(*t*)} (3)

which prevents above-baseline operation from being counted as negative loss. If *t_d_* is the disturbance onset, *t_c_* is the disturbance clearance time, and *t_r_* is the recovery time, cumulative loss over the disturbance-recovery horizon is defined as

(4) $$L_{c}=\int_{t_{d}}^{t_{r}}\max\left\{0,1-S(t)\right\}\mathrm{dt}$$

The disturbance-stage service retention is defined as

(5) $$R_{s}=\frac{1}{t_{c}-t_{d}}\int_{t_{d}}^{t_{c}}S(t)\mathrm{dt}$$

The recovery time is defined as

*T_r_* = *t_r_* − *t_c_* (6)

**Figure 7:**
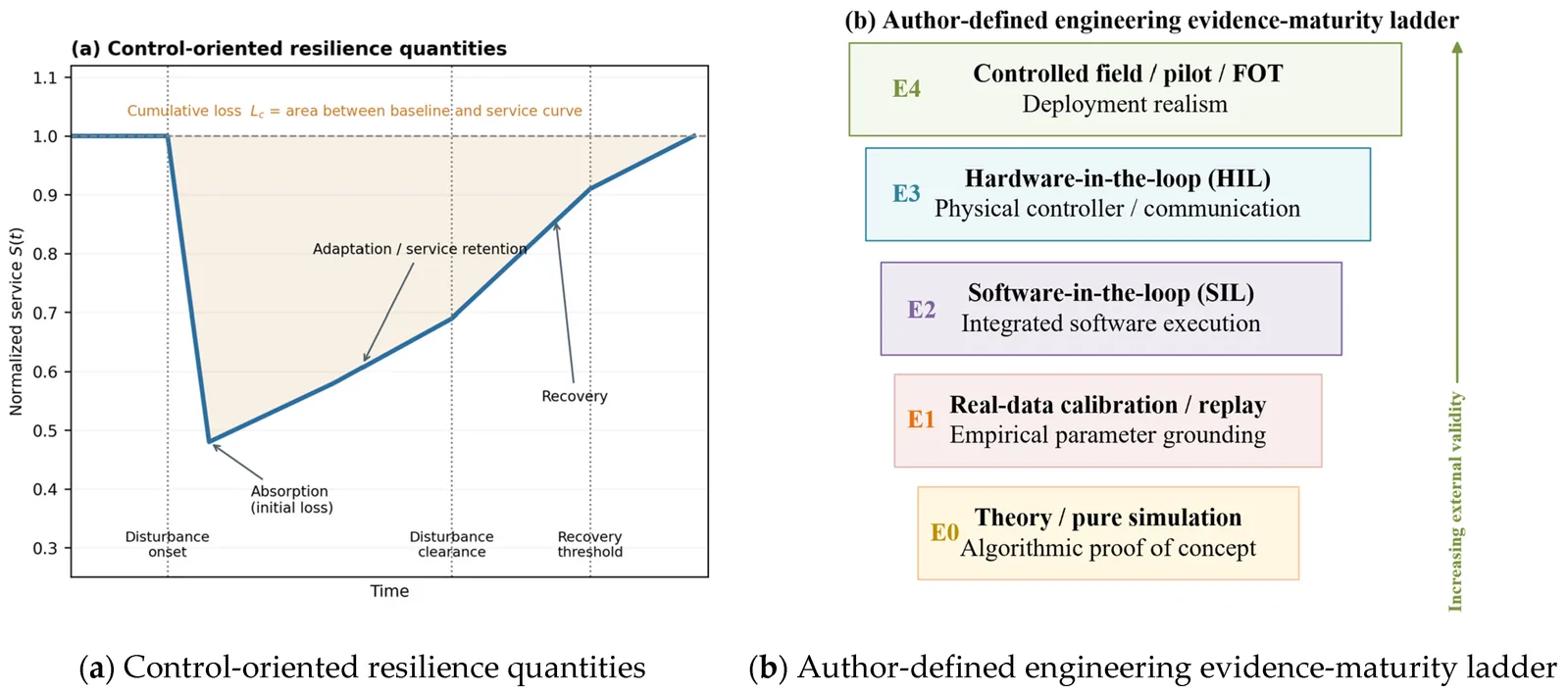
Control-oriented resilience quantities and the author-defined E0-E4 engineering evidence-maturity ladder.

The recovery instant should be determined using a threshold *η* and a persistence window Δ, so that a transient threshold crossing is not mistakenly identified as sustained recovery. The existence of *t_r_* is not assumed. If the performance trajectory does not satisfy the recovery threshold continuously for the required persistence window before the end of the observation horizon *T_obs_*, the recovery time is treated as right-censored rather than assigned an artificial finite value. In that case, cumulative loss is evaluated up to *T_obs_*, and recovery duration is reported as *T_r_* > *T_obs_* − *t_c_*. This convention also accommodates non-monotonic, discontinuous, or multi-stage recovery trajectories. Figure 7 summarizes the metrics and links them to the E0–E4 evidence ladder.

Table 6 compares the main urban road-network resilience quantification approaches, including their core applications, strengths, and limitations.

### 3.3. Disturbance Classes and Congestion Propagation

Urban road-network disturbances include natural hazards, crashes and construction, demand shocks, infrastructure/signal failures, and communication/perception failures or cyberattacks. Natural hazards may have broad spatial footprints and long recovery horizons [36], whereas crashes and work zones more often create localized capacity loss that propagates through queue spillback, forced merging, and secondary bottlenecks [37]. Large events and abnormal demand can produce similar network-scale effects through severe supply–demand imbalance.

Crashes illustrate why resilience control cannot be restricted to the failed link. Local capacity loss may spill queues through adjacent intersections, trigger gridlock, and induce rerouting that transfers congestion to distant regions. Dynamic resilience analysis therefore motivates coordinated control of upstream intersections, neighboring links, and alternative routes across the full disturbance-recovery trajectory.

Communication failures and cyberattacks propagate through a different mechanism. Malicious data, command manipulation, packet loss, and delay can spread through V2V/V2X information paths and then be amplified by vehicle and traffic dynamics. Security resilience should therefore be embedded jointly in communication and control layers rather than treated only as post-event restoration.

## 4. Mixed-Traffic Dynamics and Effective CAV Controllability

### 4.1. Vehicle Heterogeneity, Car-Following Behavior, and Penetration Effects

Connected and automated mixed traffic is intrinsically heterogeneous: vehicles differ in sensing range, communication capability, decision mechanism, and control precision. HDV car-following, lane-changing, and intersection behavior also vary with reaction time, risk preference, experience, and trust in automation, creating stochasticity that is absent from homogeneous automated-flow assumptions. CAVs can exploit onboard sensing and V2X information to execute smoother and more precise longitudinal control.

Classical car-following formulations such as the Intelligent Driver Model, Full Velocity Difference model, and Newell-type models therefore require extensions for mixed autonomy. Recent work incorporates perception errors, multi-vehicle information, nested CAV-following patterns, time-varying driving styles, and model predictive control (MPC) for smoothing speed fluctuations [38,39,40]. The key takeaway is that a CAV is not simply a short-headway vehicle; it is an information-rich, directly actuated agent whose influence depends on what it can observe and whom it can affect.

CAVs can increase capacity and stabilize traffic by shortening effective headways, suppressing perturbations, and coordinating responses, but these effects are nonlinear in penetration. At low penetration, CAVs may be spatially isolated by HDVs and have little influence over dominant traffic modes; as penetration rises, information sharing and local platoon formation expand both stability and operational controllability [41,42]. Penetration should therefore be interpreted as potential control capacity, not realized resilience capacity.

**Figure 8:**
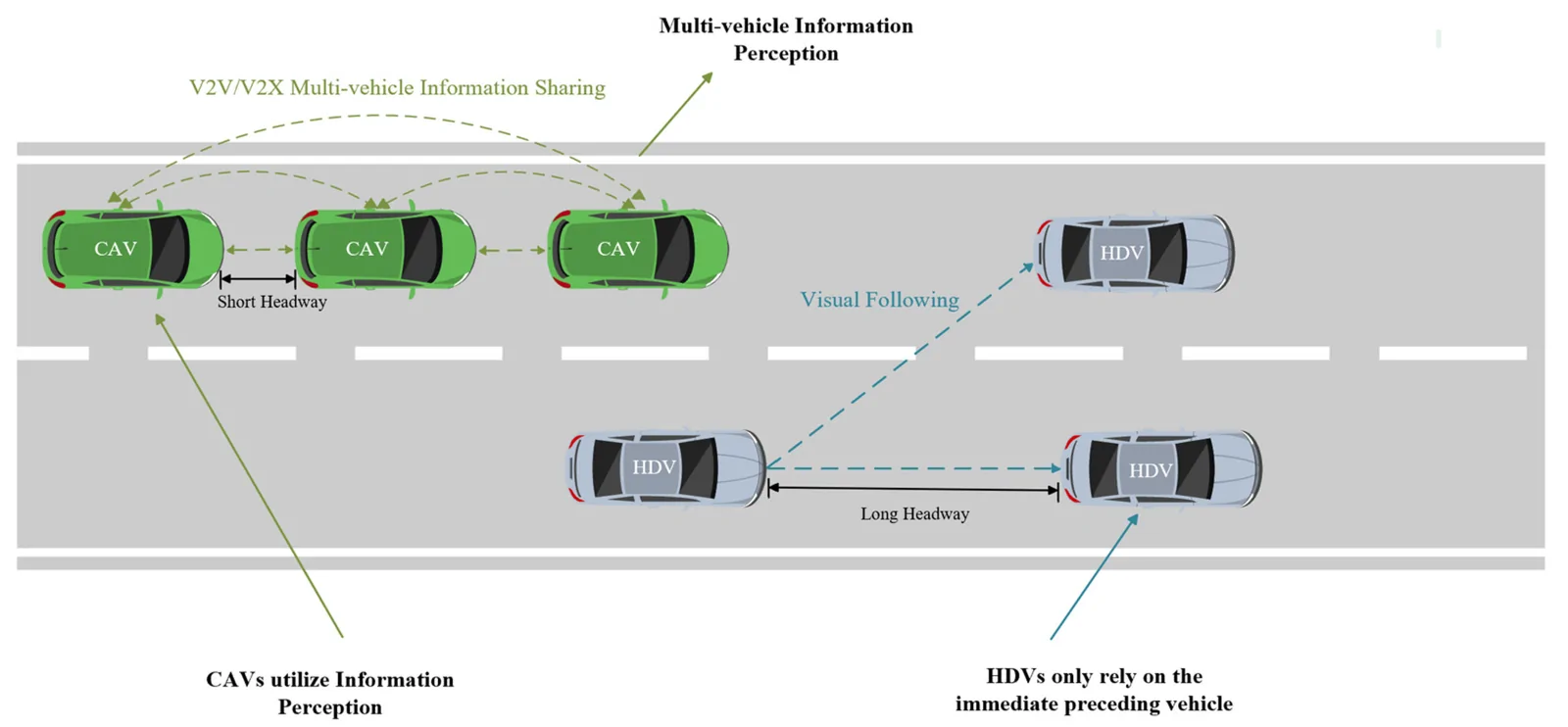
Information asymmetry and effective controllability in mixed CAV-HDV traffic.

Figure 8 illustrates how information asymmetry and spatial CAV availability shape effective controllability in mixed CAV–HDV traffic.

### 4.2. Mixed-Platoon Stability: CAVs as Mobile Actuators

A more fundamental question than penetration is whether a small number of CAVs can act as mobile actuators in traffic dominated by uncontrollable HDVs. Controllability analysis by Wang et al. [43] shows that the presence of a controllable vehicle does not make the complete mixed system controllable in the classical sense, although structured feedback can stabilize important traffic modes over a broad range of conditions. Effective controllability is therefore better interpreted as a function of controllable-vehicle location, observable information, communication structure, and the traffic modes that can actually be influenced. For operational reporting, we therefore recommend treating effective controllability as a multidimensional indicator rather than a universal scalar. At minimum, four quantities should be distinguished: (i) the functional CAV ratio ∅*F* = *N_F_*/*N*, where *N_F_* is the number of CAVs that are simultaneously observable, reachable, and able to execute a valid control action within the required time horizon; (ii) spatial control coverage, describing whether functionally available CAVs are located on bottleneck approaches or disturbance-propagation paths; (iii) model-based controllability/observability measures, such as the rank or conditioning of the corresponding controllability and observability matrices when a state-space representation is available; and (iv) disturbance attenuation, measured through changes in wave amplification, queue growth, or other task-specific input–output responses. These indicators should be reported together with penetration, communication state, and disturbance conditions rather than collapsed into a single context-free score.

At signalized intersections, the 1 + n mixed-platoon framework of Chen et al. [44] shows how one CAV leading multiple HDVs can affect platoon stability and control performance across demand and penetration levels. Related theoretical and simulation studies demonstrate that feedback gains, upstream/downstream information, and local communication topology strongly influence disturbance attenuation [41,42]. Field experiments by Stern et al. [45] further show that a single controlled automated vehicle can dissipate stop-and-go waves on a ring road, while controlled-scale data-driven predictive-control experiments show that one or a few CAVs can damp waves despite measurement noise, low-level vehicle uncertainty, and communication/computation delay [46]. These experiments strengthen the local actuation mechanism but do not, by themselves, establish network-scale resilience.

Penetration is only the first threshold; platoon organization and actuator placement form a second. At the same penetration rate, leader position, vehicle-type sequence, grouping rules, and information-flow topology can produce markedly different stability and capacity [47]. Network-level reasoning should therefore replace a scalar penetration rate with spatial availability: where CAVs are located, whether they lie upstream of bottlenecks or on disturbance-propagation paths, what they can observe, and which traffic modes or user groups they can influence.

### 4.3. Fundamental Diagrams, Macroscopic States, and Communication Constraints

Mixed-traffic fundamental diagrams link flow, density, and speed to vehicle composition, control capability, and communication state, bridging local vehicle dynamics to network-level assessment. Dynamic-network and mixed-traffic models show that CAV-HDV interactions affect network performance [13,48], while communication-constrained models show that delay and packet loss can reduce effective control authority and degrade both capacity and stability [49]. Resilience-oriented macroscopic states should therefore include communication availability or a functional CAV ratio rather than nominal penetration alone.

From an urban MFD perspective, Amirgholy and Nourinejad [50] model CAVs as regulators that orchestrate groups of HDVs, linking local spacing and group organization to network density and flow. Wang et al. [51] integrate information-flow topology, penetration, maximum platoon size, communication delay, and random link failure in a unified stability framework, revealing topology-dependent degradation boundaries.

Dedicated CAV lanes are likewise penetration-sensitive. At low penetration, they can underuse capacity or worsen lane imbalance, while benefits emerge only after CAV demand reaches an appropriate threshold [52]. Infrastructure allocation should therefore adapt to the penetration stage and prevailing traffic state rather than assume a fixed dedicated-facility policy.

### 4.4. Information Availability During the Transition Period

Resilience-oriented mixed-traffic control must account simultaneously for behavioral heterogeneity, spatial and temporal variability in CAV availability, and incomplete information caused by low penetration, occlusion, positioning error, communication delay, or packet loss [53,54,55]. These uncertainties affect both state estimation and the number of vehicles that are functionally controllable at any given time.

Low penetration does not imply negligible engineering value. Wang et al. [56] reconstructed traffic states from sparse trajectory data and optimized signal timing in a citywide field test in Birmingham, Michigan, showing that low-coverage trajectories can still support engineering-scale state estimation and signal optimization. Recent low-penetration mixed-traffic signal-control work further targets partial observability and model-based learning [57]. A practical transition strategy is therefore to distinguish information value from actuation value: use CAVs primarily as probes at very low penetration, target a small number of influential vehicles at intermediate penetration, and expand direct vehicle cooperation only when spatial availability and information quality justify it.

## 5. Cooperative Signal Control in Connected and Automated Mixed Traffic

### 5.1. From Fixed Timing to Regional Adaptive Control

Urban signal control has evolved from fixed-time and actuated strategies toward regional adaptive systems. Fixed timing is predictable and easy to implement but cannot react rapidly to demand shocks; actuated control responds to detector arrivals but may remain locally optimal under regional congestion. SCATS and SCOOT established the engineering basis for real-time multi-intersection coordination [58,59]. Their limitations under large networks, rapid demand variation, and compound disturbances motivate more adaptive, distributed, and disturbance-aware architectures.

### 5.2. Deep Reinforcement Learning and Multi-Agent Signal Control

Reinforcement learning (RL) learns signal policies through repeated interaction and is attractive when an accurate traffic model is difficult to obtain [60,61]. Deep Q-networks, proximal policy optimization (PPO), and related methods can represent nonlinear state-action-return relationships. Still, they also introduce challenges in training stability, safety, data efficiency, reproducibility, and out-of-distribution generalization.

Extending RL to multiple intersections introduces non-stationarity and a rapidly expanding joint state-action space. Multi-agent reinforcement learning (MARL) addresses these issues using value decomposition, actor–critic methods, and centralized training with decentralized execution (CTDE). VDN and QMIX support cooperative value decomposition [62,63], while MADDPG and MAPPO use richer training information to reduce non-stationarity [64,65]. In connected traffic, the same architecture can coordinate signal agents with vehicle agents [66]. Still, the resulting communication and action dimensions become part of the engineering problem rather than merely an algorithmic detail.

From a resilience perspective, signal-control research is moving from robustness testing under abnormal inputs toward explicit disruption objectives. DRL controllers have been stress-tested under demand surges, incidents, and sensor failures [67]. Shang et al. [68] link adaptive signal control with day-to-day dynamics under disruption, Xiang et al. [69] directly optimize urban road-network resilience, and targeted evidence includes robust adaptive control under low V2I penetration [70] and delay-aware state compensation for multi-intersection control [71]. The remaining gap is cross-layer: communication degradation, recovery-phase switching, and safe fallback are rarely optimized jointly with CAV-HDV actuation.

## 6. Signal–Vehicle Coordination from Intersections to Corridors

### 6.1. Model-Based, Adaptive, and MPC Signal-Trajectory Coordination

Connectivity gives signal controllers access to vehicle positions, speeds, and expected arrival times, improving state estimation and arrival prediction. At low penetration, connected vehicles can function as mobile probes and cooperative sensing resources for multi-intersection systems [72]. As effective controllability grows, vehicles become active actuators: coupled signal–vehicle control simultaneously adjusts signal timing and CAV trajectories so that signal-defined passage windows and vehicle arrival-time shaping form a bidirectional loop [73,74].

MPC is attractive because it can explicitly represent vehicle dynamics, acceleration/speed limits, signal phases, and safety constraints. Still, online complexity increases quickly with vehicle count, horizon length, and network scale. Hierarchical and decomposition-based formulations address this burden. Zou et al. [75] combine upper-level platoon-based mixed-integer signal optimization with lower-level distributed MPC; Liu et al. [76] formulate hybrid optimal control with continuous acceleration and discrete signal switching; and He et al. [77] combine back-pressure signal control with discretized CAV trajectory control at low penetration, Lyapunov analysis, and two months of connected-bus operational data. HIL evidence at a queue-aware signalized intersection [78] and more recent mixed-traffic testbed validation of vehicle–traffic-light cooperation [79] strengthen the feasibility chain, but repeatable disturbance-and-recovery validation remains limited.

Recent work also models HDV uncertainty explicitly. Hierarchical stochastic signal optimization and robust trajectory control use chance constraints for uncertain HDV departure times and robust MPC for following disturbances [80]. This is a meaningful improvement over perfectly known HDV trajectories, although validation remains predominantly simulation-based.

### 6.2. Deep-Reinforcement-Learning-Based Joint Control

When demand, driving behavior, and penetration vary rapidly, model mismatch can degrade optimization and MPC. Deep reinforcement learning (DRL) therefore learns signal and vehicle decisions jointly, using traffic-state prediction, fuzzy feature fusion, and control of only a selected subset of CAVs to reduce vehicle-level action dimensionality [81,82,83].

**Figure 9:**
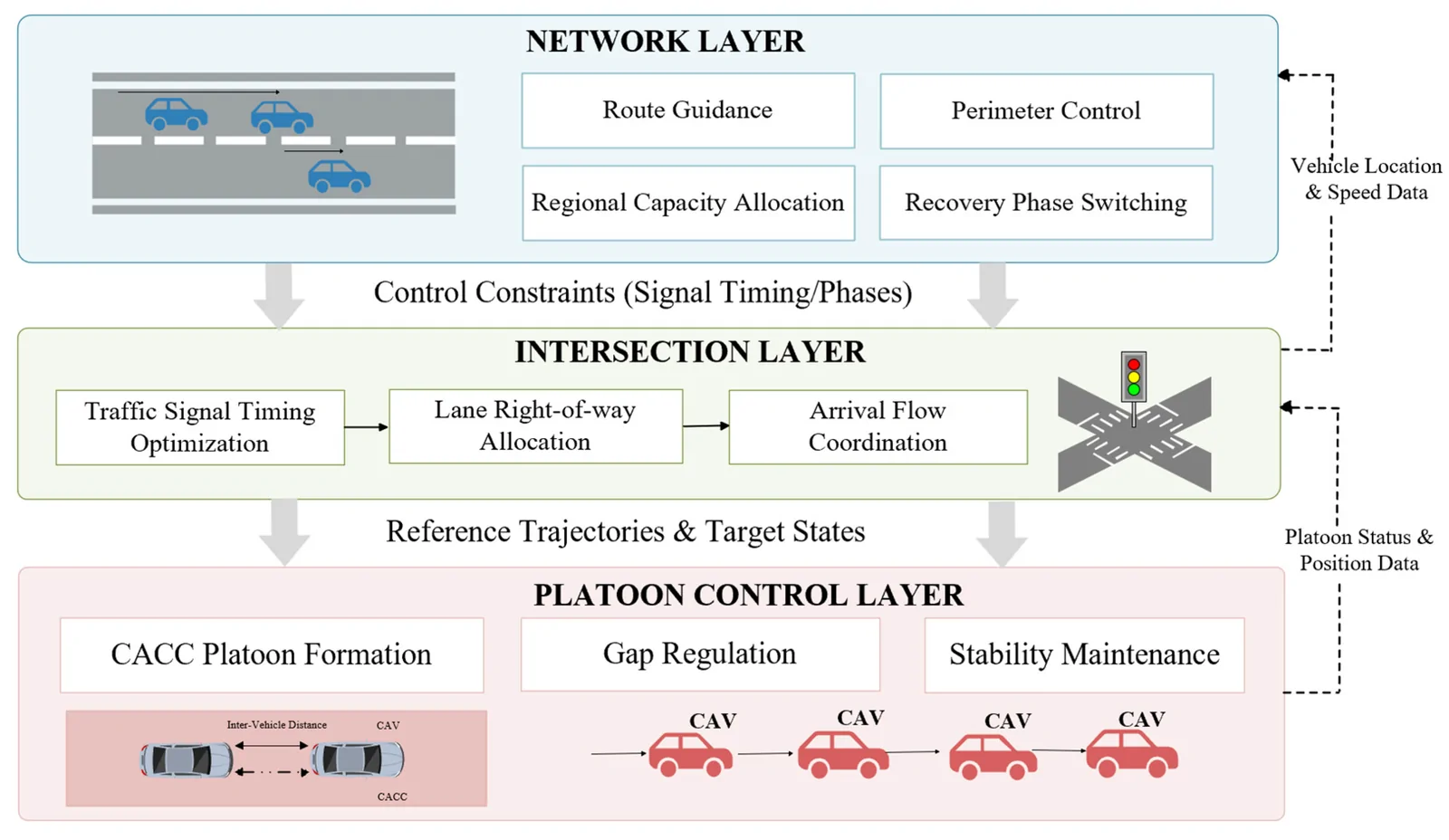
Hierarchical signal–vehicle–route cooperative-control architecture for urban network resilience. Solid arrows indicate control/reference or intra-layer information transmission, whereas dashed arrows indicate bottom-up vehicle/platoon state feedback.

CoTV coordinates traffic signals with a limited set of key CAVs, reducing vehicle-level action dimensionality [83]. Joint DRL and hierarchical PPO formulations combine discrete signal decisions with continuous trajectories or accelerations under delay, energy, and comfort objectives [84,85], while model-guided RL injects traffic knowledge into learning [86]. Other frameworks optimize signal timing together with lane configuration, vehicle sorting/platooning, or trajectories [87]; asynchronous MARL separates high-frequency vehicle planning from lower-frequency signal decisions [88]; and vehicle–road–cloud actor–critic architectures distribute training and execution across cloud, edge, and vehicles [89]. Emerging hierarchical network-signal/trajectory optimization extends this logic to larger connected networks [90]. Collectively, the field is moving from signal-only agents toward hybrid signal–vehicle actuation, but explicit optimization of absorption, service retention, and recovery is still uncommon [91,92]. Representative intersection- and corridor-scale coordination methods and their evidence boundaries are summarized in Table 7. Figure 9 synthesizes these mechanisms into a hierarchical signal–vehicle–route cooperative-control architecture.

### 6.3. Platoon Cooperation and Corridor-Level Control

Platoon control can improve capacity, fuel economy, and traffic-flow stability through coordinated acceleration and reduced headways. In mixed traffic, however, CAV-led platoons interact with uncontrollable HDVs, changing information topology, and communication delay. Distributed MPC and distributed learning are therefore used not only to coordinate CAV actions but also to attenuate disturbances that originate outside the controllable subset.

Beyond string stability, mixed-platoon control supports eco-driving through successive signalized intersections [96], safety assessment of cooperative platooning [97], and distributed DRL for damping oscillations under partial CAV control [98]. Corridor studies jointly consider penetration, saturation, travel time, and fuel use [95], while recent multi-level signal–vehicle approaches extend hierarchical coordination across arterial intersections [90,94]. The evidence confirms a direct mechanism linking vehicle-level shaping to corridor control, but most objectives still emphasize normal-operation efficiency, energy, and safety rather than recovery.

### 6.4. Lane Allocation, Lane Changing, and Cooperative Driving

Link-level control forms the interface between microscopic intersection decisions and macroscopic network management. Dynamic CAV-lane allocation, lane selection, cooperative lane changing, and infrastructure-assisted driving can use roadside sensing and V2X to modify approach order and local conflict structure [93,99]. When coupled with signal control, lane use can adapt to penetration, demand, and bottleneck state.

Longitudinal–lateral coordination becomes particularly important during local blockages. A two-level framework that couples lane-change decisions with speed-trajectory optimization uses a small number of CAVs to relieve blockage in shared lanes and smooth through movements [100]. This evidence indicates that resilience-oriented CAV actuation should not be reduced to longitudinal speed control when disturbances involve lane loss, forced merging, or spatially localized bottlenecks.

### 6.5. Integrating Safety, Efficiency, and Implementability

Efficiency-only learning can produce rapid phase switching, excessive acceleration, or unsafe headways. Cooperative control should therefore move from soft safety penalties in reward functions toward explicit safety constraints in the feasible control set. Recent studies use surrogate safety indicators, safe eco-driving RL, and safe MARL for connected intersections [101,102,103].

For deployment, learned actions should also respect minimum and maximum greens, yellow/all-red transitions, anti-starvation rules, speed and acceleration limits, minimum headway, conflict checking, and fallback logic. Mixed-platoon safety evidence similarly shows that benefits depend on interpretable constraints and reliable execution, not on policy performance in nominal simulation alone [104].

## 7. Network-Level Cooperative Control and Explicit Resilience Optimization

### 7.1. Regional Signal Coordination and Dynamic Routing

At regional scale, the number of control variables, real-time constraints, and competing objectives grows sharply. Connected-vehicle states can support network-wide signal optimization [105], while route guidance redistributes demand spatially. Their coordination is particularly important after incidents because signals allocate local right-of-way whereas routing exploits residual capacity. Trinh et al. [106] couple multi-intersection signal control with rerouting in mixed traffic. Complementary evidence on network resilience under CAV deployment [107] indicates that penetration can change both disruption propagation and recovery opportunities. However, most studies still evaluate network performance without a common set of disturbance-recovery metrics.

System-optimal rerouting can also impose longer routes or higher costs on particular users. Network redundancy, alternative-route capacity, spatial heterogeneity, and service equity therefore need to be considered jointly [107,108]. A resilience-oriented controller should avoid restoring one region by exporting congestion or risk to another vulnerable region or user group.

### 7.2. Scalable Network-Level Signal-Trajectory Coordination

Large urban networks can contain hundreds of signal controllers and thousands of candidate vehicle agents, making fully centralized optimization difficult under real-time constraints. Scalable approaches therefore decompose the problem spatially, temporally, or by control object. Distributed RL, parameter sharing, graph representations, and hierarchical signal-trajectory architectures can reduce the dimension of local optimization or policy-execution problems, although preservation of closed-loop coordination, stability, and convergence remains formulation dependent. This review does not assume a universal fixed-point or convergence property for hierarchical multi-agent coordination. If an iterative scheme is represented abstractly as *z^k^*^+1^ = *F*(*z^k^*), a contraction bound such as ‖*F*(*z*_1_) − (*F*(*z*_2_)‖ ≤ *κ*‖*z*_1_ − *z*_2_‖ with 0 ≤ *κ* < 1 would be a sufficient condition for convergence to a unique fixed point. The reviewed signal–vehicle–route literature does not establish such a common bound across heterogeneous architectures. Therefore, convergence claims are treated as method-specific and are accepted only when supported by the original study’s theoretical analysis or empirical stopping/feasibility evidence. A central engineering question is not whether a decomposition improves nominal throughput, but whether it preserves the information and control pathways needed for recovery when parts of the network or communication layer are degraded.

Likewise, vehicle-level string stability or wave-suppression performance should not be assumed to remain unchanged after regional signal, routing, or perimeter feedback is closed around the local subsystem. Such extrapolation requires either an explicit interconnection analysis—for example, a suitable small-gain or time-scale-separation argument—or joint closed-loop testing. In the absence of such evidence, this review restricts local stability claims to the spatial and control scale at which they were originally demonstrated.

Model-based multi-scale decomposition provides a complementary path. Gao and Ban couple fast vehicle control with slower signal control in a multi-scale framework [109], and related work coordinates intersection- and network-level decisions using constrained or hierarchical optimization [110,111,112]. The emerging network-scale signal-trajectory literature, including hierarchical multi-agent formulations [90], is important because it makes the local-to-network mechanism computationally explicit. Yet network-wide disturbance recovery, communication failure, and field validation are still rarely combined in one experiment.

Explicit resilience optimization remains the principal boundary. Most signal, trajectory, and routing studies optimize delay, throughput, emissions, energy, or safety under nominal or mildly perturbed conditions. By contrast, resilience requires controllers to manage the full performance trajectory: limit the initial drop, maintain acceptable service under constrained capacity, and accelerate recovery without exporting excessive congestion or risk to adjacent regions or user groups. Recent urban road-network frameworks provide dynamic and zonal resilience metrics [113,114], while resilience-enhanced road-traffic control has begun to formulate control directly around disturbance response [115]. These developments provide a stronger bridge between traffic-control objectives and resilience theory, but they have not yet converged on common mixed-CAV benchmarks.

Cooperative control and transportation resilience developed largely as parallel research streams, but direct convergence is now visible. Adaptive signals and DRL have been used to enhance urban road-network resilience under capacity loss [68,69], perimeter control has been designed explicitly for disrupted urban networks [116], and mixed-traffic signal–vehicle control is expanding from isolated-intersection efficiency toward multi-scale networks and signal-routing coordination [86,106,110,111,112].

Yue et al. [117] integrate microscopic CAV driving behavior, macroscopic mixed-traffic assignment, incident bottlenecks, and dynamic signals in a cooperative incident-management framework. This directly addresses the cross-scale chain linking controllable CAV resources, incident disturbance, and network allocation/signal response. Nevertheless, most reported outcomes focus on stability, delay, or generic network performance rather than a unified online objective combining service retention, cumulative loss, recovery time, communication degradation, and recovery-phase control.

Complementary resilience evidence includes traffic-responsive signal control under hazardous-material disruptions [118], learning-assisted network recovery scheduling under uncertainty [119], and post-congestion recovery optimization for mixed-traffic networks with uncertain demand [120]. Multi-agent traffic control has also considered resilience-by-design under attacks [121], while MFD-based perimeter control has been made resilient to deception and denial-of-service attacks [122]. Recent work on network fragility strengthens the theoretical basis for testable resilience claims [123]. The common lesson is that resilience is moving from post hoc assessment into the control objective, but a lower average delay under one disruption should not be generalized as universal resilience improvement.

### 7.3. Multi-Scale State Representation and Hybrid Action Spaces

Incident queues propagate through network topology and can generate long-range dependencies through spillback and rerouting. Single-intersection states are therefore insufficient. Fixed-neighbor aggregation may miss dynamic congestion correlations, while fully connected encoders may sacrifice physical interpretability. Graph-based representations preserve road adjacency, and attention mechanisms can capture longer-range dependencies. Still, their value should be tested under cross-network, cross-disturbance, and communication-degradation conditions rather than inferred from normal-operation delay reductions alone.

The action space is also inherently hybrid: signal decisions are discrete, whereas vehicle speed, acceleration, and trajectories are continuous. Treating every CAV as an independent agent causes action growth that is difficult to scale, while signal-only control discards the flow-shaping capability of controllable vehicles. Key-vehicle selection, hierarchical decomposition, distributed optimization, and lane-signal coordination offer practical ways to compress the hybrid action space without abandoning cross-scale actuation.

Computational complexity also differs substantially among control families. Mixed-integer signal-trajectory optimization can grow rapidly with the number of binary signal/sequence decisions and the prediction horizon; continuous MPC shifts the burden toward state dimension, horizon length, and solver iterations; centralized MARL faces growth in joint state-action spaces and training sample demand; and hierarchical, graph-based, parameter-sharing, or key-vehicle approaches reduce local action or communication dimensions at the cost of additional coordination and synchronization. Because asymptotic complexity is highly implementation-dependent, future comparisons should report network size, controlled vehicles/intersections, state/action dimensions, prediction horizon or model size, coordination iterations, per-decision wall-clock latency, computing hardware, and deadline misses.

## 8. Enabling Technologies and Engineering Validation

### 8.1. V2X Communication, Cybersecurity, and Graceful Degradation

V2X communication underpins signal–vehicle coordination, so latency, synchronization, reliability, bandwidth, coverage, information integrity, and computation delay directly affect control authority. Recent V2X survey work shows that cooperative outputs are useful only when shared evidence remains timely, trustworthy, and actionable under realistic wireless conditions [24]. Similarly, cellular-V2X reviews identify access reliability and resource constraints as deployment-limiting factors [124,125,126]. Traffic-control studies should therefore avoid treating communication as a lossless transparent channel. Communication can itself become a control variable: traffic actions change spatial message demand and contention, while communication allocation changes observability, coordination range, and which vehicles are functionally controllable. This motivates joint traffic-communication design and condition-aware fallback rather than testing a nominal controller against communication impairment only after training.

A useful abstraction is a control-quality contract: every cooperative state or command should be interpreted together with its freshness, synchronization error, delivery reliability, communication load, spatial reachability, trust/integrity status, positioning or perception uncertainty, and end-to-end computation latency. This idea is analogous to communication-aware evidence contracts used in recent V2X safety research [24], but here, the contract is tied directly to feasible control action. Task- or semantic-oriented communication can further reduce redundant exchange by prioritizing information that changes queue estimation, arrival shaping, conflict resolution, or safety margins rather than transmitting all available data [127]. The control benefit, however, should be demonstrated under fixed communication budgets and explicit degradation curves; reduced bandwidth alone is not evidence of network resilience.

Direct platoon evidence shows that communication loss changes string-stability boundaries under multi-predecessor information topologies [128], and experimental data show increased motion variability under communication delay [129]. Reliable cooperative control can preserve platoon stability under cyberattacks [130]. Broader CAV studies now evaluate resilience to cyber–physical disruptions [131], and survey work systematizes attack detection and resilient control from a vehicle-dynamics perspective [132]. Real-time platoon frameworks that combine attack detection with compensation [133] further illustrate the shift from ‘detect then stop’ toward detect–isolate–degrade–recover architectures. These results are important but mostly local; they do not yet establish how communication impairment changes network-level service retention when signals, routes, and distributed CAV actuators interact.

**Figure 10:**
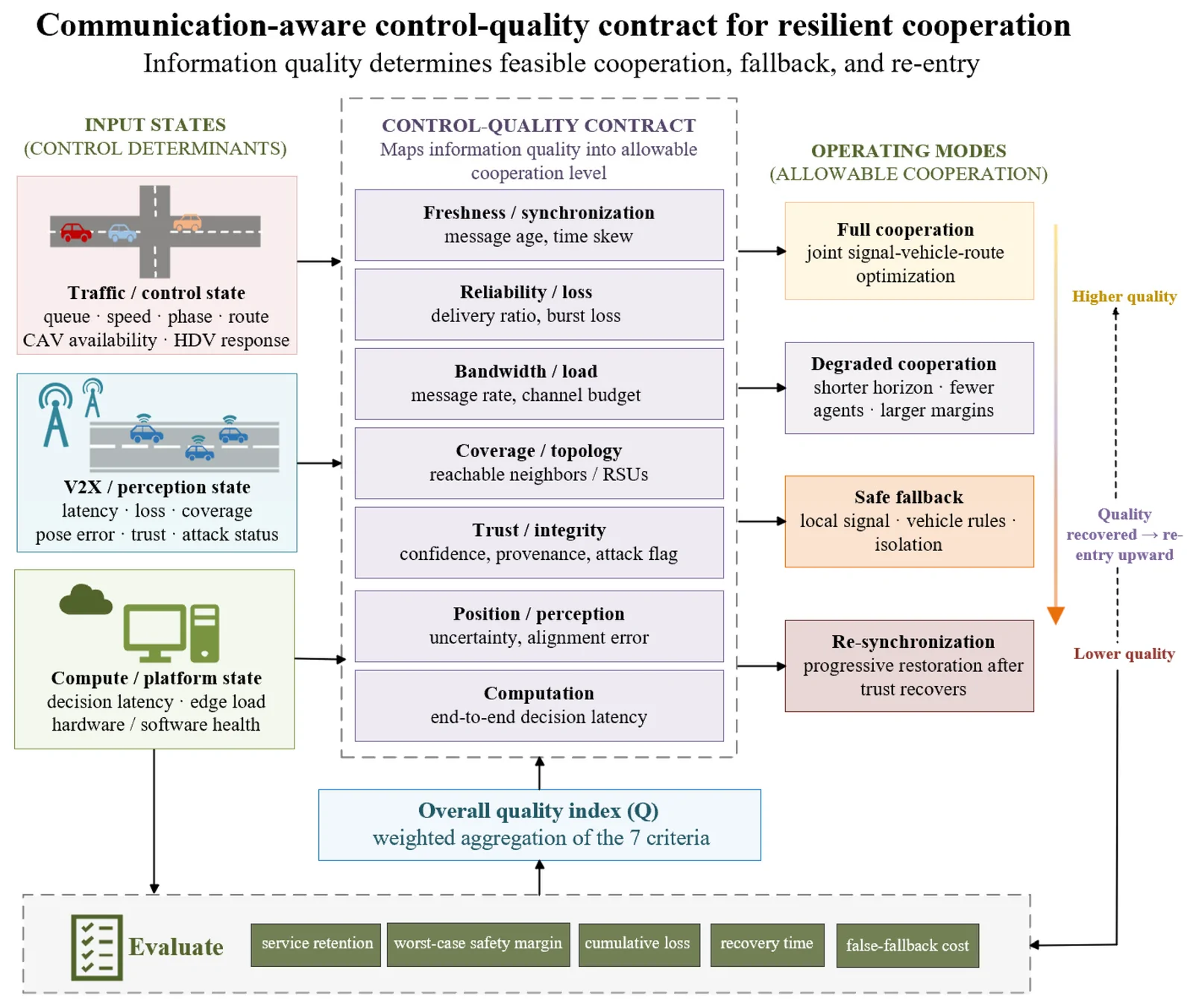
Communication-aware control-quality contract linking traffic/V2X/compute states to cooperation, fallback, and re-synchronization.

A practical resilience loop should therefore follow detection–isolation–degradation–recovery. When freshness, reachability, or trust falls below predefined thresholds, the controller should shorten the cooperation horizon, reduce the number of coupled agents, increase safety margins, down-weight suspect information, or revert to local signal/vehicle rules. As information quality recovers, cooperation should be reintroduced progressively rather than instantaneously. To prevent chattering near a switching boundary, the contract should use hysteretic rather than single-threshold transitions. For a monitored quality variable *q*, a degraded mode is entered when *q* crosses a degradation threshold *θ_down_* for at least a persistence interval Δ*_down_*, whereas return to the higher cooperation mode requires satisfaction of a stricter recovery threshold *θ_up_* for Δ*_up_*, together with a minimum dwell time. The two thresholds need not be universal constants; they should be selected according to the safety margin, prediction horizon, estimator uncertainty, and communication requirements of the control task. Communication reachability alone does not guarantee state observability. For a local linearized model with state dimension *n_x_*, an observable-state indicator may be reported as *ρ*_O_ = *rank*(O)/*n_x_*, where O is constructed only from measurements that remain available and valid under the current sensing and communication state. Similarly, *ρ_c_* = *rank*(*C*)/*n_x_* may be used as a controllability indicator when the corresponding actuation model is available. Rank-based measures are only one option; for nonlinear or learned systems, state-estimation uncertainty or empirical observability measures may be more appropriate. Figure 10 summarizes this control-quality contract; Table 8 and Table 9 summarize the engineering bottlenecks and the corresponding control-quality/stress-test requirements, respectively. Evaluation should include service retention, worst-case safety margin, false-fallback cost, cumulative loss, and recovery time in addition to attack-detection accuracy or packet-delivery ratio.

### 8.2. Self-Sensing UHPC as a Physical-Infrastructure Sensing Layer

The sensing problem in resilient mixed traffic is not limited to vehicles and communications. A crash, bridge-deck or joint deterioration, local cracking, or other structural degradation can reduce usable capacity before its full effect is evident in speed, queue, or probe-trajectory measurements. Intrinsic self-sensing UHPC offers a complementary physical-infrastructure sensing layer because conductive fillers or aggregates create electrical networks whose resistance, resistivity, impedance, or fractional change in resistance (FCR) varies with stress, strain, cracking, and damage. The recent state-of-the-art review by Song et al. [142] shows that this field has moved from material formulation toward integrated sensing components, while also emphasizing unresolved issues in scale fabrication, cost, long-term stability, data acquisition, transmission, and structural integration. For this traffic-control review, the relevant point is therefore not that UHPC replaces conventional detectors, but that it may add a physically colocated measurement of infrastructure condition to the cyber–physical state used by digital twins and resilience controllers.

The relevance to traffic control is therefore a translation problem rather than a material-selection problem. Conceptually, the required chain is electrical observable → structural state with confidence → usable-capacity/lane-availability state → traffic-control action.

For example, a persistent electrical change that has been independently calibrated as a crack- or damage-state indicator could update the capacity prior of an affected lane or link in the digital twin; this update could then trigger a conservative speed limit, lane restriction, signal adjustment, rerouting, perimeter-control action, or CAV trajectory constraint. Conversely, when the material signal is uncertain because of temperature, moisture, electrode condition, drift, or communication failure, it should not directly drive traffic control and should instead be down-weighted or rejected by the state-estimation layer.

The 12-paper bridge set demonstrates several routes for constructing responsive conductive networks. Carbon-based fillers such as nanocarbon black, graphene/CNT, and hybrid carbon black/carbon nanofiber systems improve piezoresistive sensitivity by forming conductive pathways and modifying percolation behavior [143,144,145,146]. Several studies further combine material characterization with structural analysis to identify candidate sensing locations and evaluate the relationship between conductive response and mechanical states [145,146].

Metallic, recycled, and hybrid conductive constituents provide additional pathways for improving electrical responsiveness, structural compatibility, and sustainability. Studies on steel-slag UHPC, recycled-carbon-fiber UHPC, cementless conductive UHPC, and hybrid steel-fiber-based conductive networks demonstrate that alternative reinforcement strategies can generate measurable electrical responses while maintaining structural functionality [147,148,149,150,151]. These studies broaden the material basis for infrastructure-state sensing, although their direct translation into traffic-control decisions still requires intermediate state-estimation and validation layers.

Environmental factors remain critical because moisture, temperature, ionic conduction, electrode layout, loading history, and aging can modify the electrical response. Therefore, laboratory FCR-strain relationships cannot be directly transferred into traffic-control decisions without calibration, uncertainty estimation, and validity assessment [152,153].

**Figure 11:**
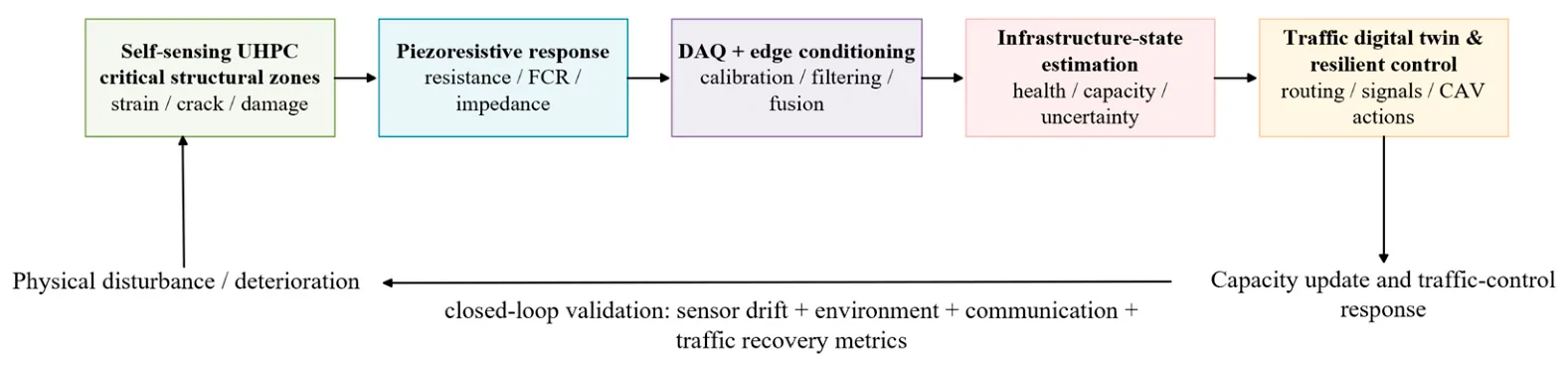
Material-integrated infrastructure sensing as an upstream physical-state layer for digital-twin and resilient mixed-traffic control.

Figure 11 positions self-sensing UHPC in the broader resilience loop. A practical architecture would place self-sensing material only at structurally critical zones—for example, bridge joints, deck regions, repair interfaces, or other capacity-critical components—and condition the electrical signal at a local data-acquisition/edge layer. The resulting health or capacity estimate could then enter a traffic digital twin together with probe trajectories, roadside detection, signal status, and V2X state. Only after structural thresholds and uncertainty are validated should that information modify lane availability, link-capacity priors, speed limits, routing, perimeter control, or signal/CAV coordination. None of the 12 papers demonstrates this complete closed loop; the proposed coupling is therefore a bounded research direction rather than established CAV-resilience evidence.

For transportation resilience, the key research task is not to maximize material gauge factor in isolation but to define a trustworthy interface between the structural sensor and the traffic controller. Table 10 summarizes the targeted bridge evidence, while Table 11 converts the proposed interface into control-relevant observables and validation requirements. The material layer should report both the estimated physical state and its confidence/validity flag; the traffic layer should use the signal only when it changes a decision-relevant quantity such as usable capacity, lane availability, safe speed, or disturbance persistence. This preserves the review’s central control-quality principle: a sensor is valuable only when its information is timely, calibrated, decision-relevant, and accompanied by a safe fallback.

### 8.3. Edge–Cloud Coordination

Control loops operate on different time scales. Vehicle trajectory and safety control should remain on the vehicle or roadside edge; intersection/corridor coordination can run at a nearby edge layer; and slower network policy updates, cross-region data fusion, and offline training can be placed in the cloud. This hierarchy reduces the risk of making second-level or safety-critical decisions dependent on high-latency remote links.

In addition, 5G/6G and edge intelligence can reduce backhaul load and coordination delay, but they also introduce asynchronous edge states, policy-consistency problems, computation-resource allocation, and new cybersecurity exposure. Edge–cloud coordination should therefore be co-designed with distributed MARL, communication budgets, and fallback logic. Because direct end-to-end evidence for edge–cloud–vehicle resilience in mixed urban traffic remains fragmented, this review treats the architecture as a design implication rather than an established deployment standard.

### 8.4. MARL, Digital Twins, and Sim-to-Real Transfer

MARL is increasingly used for multi-intersection and signal–vehicle coordination, but real roads cannot support unconstrained online trial-and-error. The 2026 scoping review by Ortega et al. [25] shows that CAV-resilience evidence remains dominated by simulation and analytical studies, with much less pilot, field, or field-operational-test evidence. SUMO, CARLA, and related environments enable efficient stress testing, yet domain gaps remain in car-following, lane changing, driver behavior, demand, communication quality, and equipment failure. Multi-intersection engineering platforms [23] and emerging mixed-traffic testbeds [79] demonstrate progress, but field operation alone is not equivalent to resilience validation under controlled, repeatable disturbance.

Digital twins can be calibrated using floating-car trajectories, signal logs, roadside detectors, communication data, and, where structurally validated, infrastructure-condition measurements from embedded or material-integrated sensors. X-in-the-loop methods connect virtual and physical automated-vehicle testing [141], and HIL studies demonstrate how connected-vehicle applications can be exercised against physical controller hardware [78]. A defensible validation chain is therefore real-data calibration → high-fidelity digital twin → SIL → HIL → controlled pilot/field testing. Scaled physical mixed-traffic experiments [79] and data-driven wave-suppression experiments [46] show that some work is moving beyond pure simulation. Still, repeatable urban-network field operational tests under communication and physical disruptions remain rare, and the self-sensing UHPC evidence in Section 8.2 has not yet been integrated into such traffic-control validation chains.

Engineering maturity should be judged by reproducibility and external validity as well as average performance. Learning-based controllers should report multiple random seeds, variance or confidence intervals, worst-case performance, per-decision computation time, hardware, and whether training and test networks, demand, driver behavior, and communication parameters are independent. Cross-network tests, domain randomization, and communication-degradation stress tests are needed to distinguish scenario-specific optimization from transferable control.

### 8.5. Benchmark and Evaluation Infrastructure for Closed-Loop Resilience

A major transferable lesson from recent V2X survey practice is that end-to-end claims require an evaluation infrastructure aligned with the claimed system loop, not only a collection of algorithm-level metrics [24]. For mixed-traffic resilience control, the benchmark unit should therefore be a complete disturbance episode that couples traffic composition, physical disturbance, communication/perception conditions, computation limits, control actions, and the recovery trajectory. A reproducible run should preserve enough provenance to answer who or what was observable and controllable, which information was available when each decision was made, and how the network returned to service.

The E0–E4 ladder becomes more useful when tied to specific evidence capabilities. Analytical or microscopic simulation can establish mechanism plausibility and broad sensitivity; traffic-communication co-simulation can expose cross-layer degradation; real-data replay and calibrated digital twins can test realism and domain shift; SIL and HIL can reveal interface and timing failures; and controlled pilots or field-operational tests can establish whether the full control loop remains safe and effective under operational constraints. Table 12 summarizes these roles and their claim boundaries.

**Figure 12:**
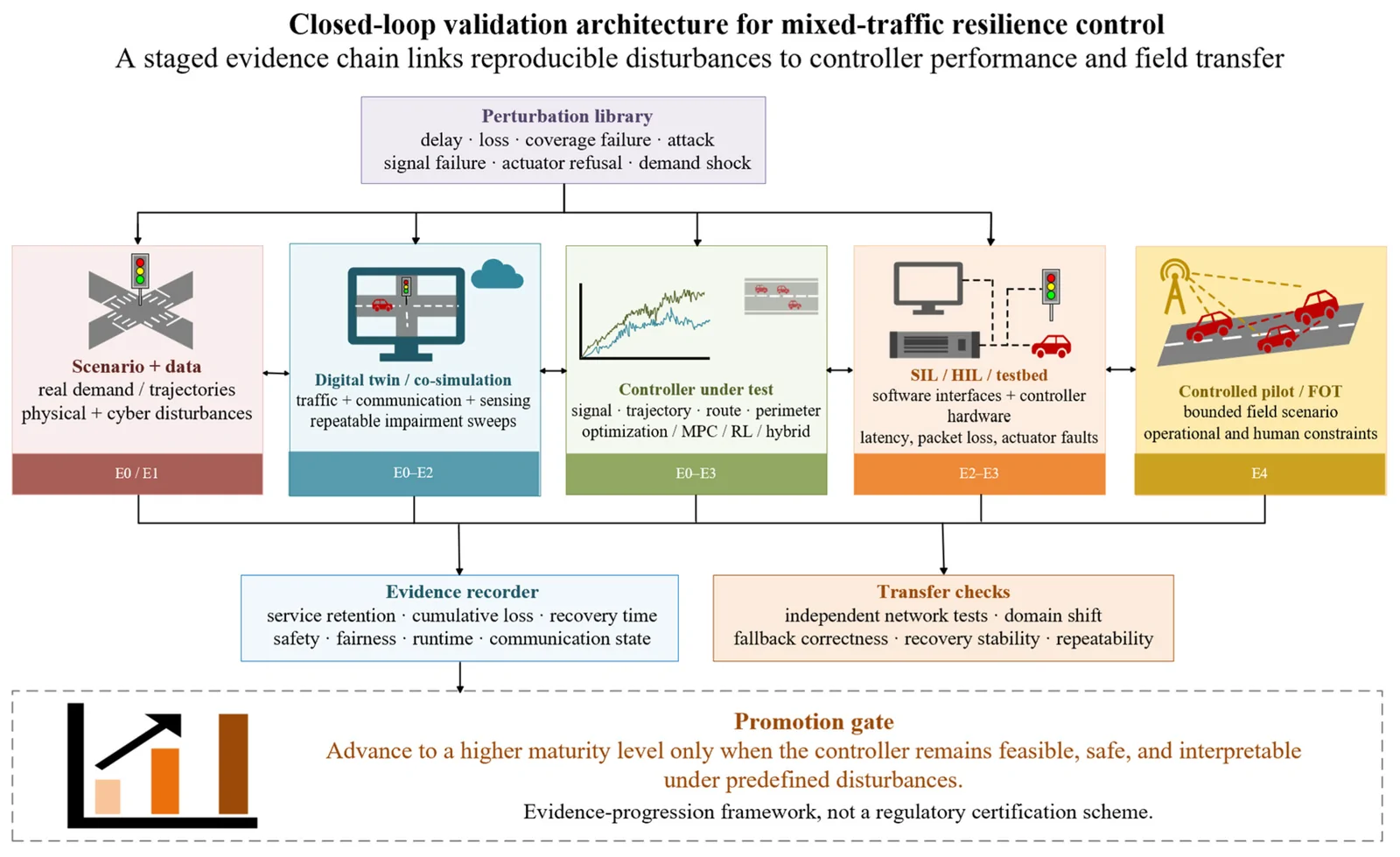
Closed-loop validation architecture linking disturbance libraries, co-simulation/digital twins, SIL/HIL, and field evidence.

Figure 12 expresses the same idea as a closed-loop validation architecture. The promotion criterion between maturity levels is not a higher average reward but retained feasibility, safety, interpretability, and recovery under predefined disturbances. This prevents a field-connected platform from being mistaken for resilience evidence when repeatable abnormal conditions have not challenged the mechanism.

### 8.6. Cross-Layer Lessons Learned

Lesson 1—Information quality and infrastructure observability are part of the control state. Latency, loss, coverage, trust, perception error, computation timing, and the availability and calibration of physical infrastructure-condition measurements determine functional controllability and should enter state estimation, action selection, or fallback logic rather than appear only in a sensitivity appendix.

Lesson 2—Joint control is not automatically resilience control. Coupling signals and vehicles can improve delay or energy while leaving disturbance absorption and recovery unchanged; resilience requires explicit disturbance-stage objectives and a definition of recovery.

Lesson 3—Decomposition should follow both physical scale and failure locality. Fast safety and trajectory loops should remain local, corridor coordination can operate at the edge, and slower routing/perimeter policies can act regionally, with fallback designed so failure at one layer does not turn off all layers.

Lesson 4—Engineering evidence should be condition-tagged. Every reported gain should be interpretable alongside traffic composition, CAV spatial availability, disturbance severity, communication state, computation budget, baseline information access, and E0-E4 maturity so transfer claims can be bounded. Material-integrated sensing requires the same discipline: reported strain/crack sensitivity should be tagged by moisture, temperature/curing, electrode and acquisition configuration, polarization treatment, loading regime, specimen scale, and structural integration state before it is propagated into traffic-capacity or routing decisions.

## 9. Cross-Scale Synthesis, Research Agenda, and Limitations

### 9.1. Cross-Scale Mechanism and Evidence Strength

The evidence supports a bounded cross-scale mechanism rather than a universal penetration-benefit relationship. Nominal CAV penetration becomes effective controllability only through spatial vehicle availability, platoon organization, HDV response, information quality, and decision timing. Effective controllability then modifies link and intersection states through wave suppression, arrival shaping, conflict management, and queue regulation. Only when those local effects are coupled with regional signals, routing, perimeter control, or residual-capacity allocation can they plausibly reduce network cumulative loss and recovery time. The communication-aware contract in Figure 10 adds a further condition: the set of functionally controllable vehicles and feasible joint actions changes dynamically as latency, coverage, trust, and computation quality change. Figure 11 adds a complementary upstream concept in which validated structural self-sensing can update physical infrastructure state or capacity before that information is fused into the traffic digital twin. Because the bridge-set literature has not yet demonstrated this material-to-control link, it should be treated as an observability extension rather than evidence of a resilience benefit. This mechanism explains why single-intersection, ideal-communication, nominal-penetration, or material-level sensing results should not be extrapolated directly to network resilience.

Evidence is comparatively mature for mixed-platoon stability, local intersection efficiency, and vehicle-level wave suppression, supported by theory, controlled experiments, selected field experiments, and operational data. Explicit signal-control resilience is emerging, and the targeted candidate update adds low-penetration signal control [57], delay-aware multi-intersection control [71], physical testbed validation [79], hierarchical network signal-trajectory coordination [90], network-resilience assessment [107,113,114], and communication/cyber-resilience evidence [131,132,133]. The weakest evidence remains city-scale closed-loop experiments that simultaneously include mixed CAV/HDV actuators, regional signals, routing/perimeter or capacity reconfiguration, degraded information, and unified recovery metrics. The central conclusion is therefore conditional: local controllability contributes to network recovery only when state, control, metric, disturbance, and validation conditions are made explicit. Table 13 summarizes the synthesis, relative evidence strength, and major evidence boundaries for RQ1–RQ4.

### 9.2. Toward a Unified Signal–Vehicle–Route Closed Loop

Future systems should move from loosely coupled upper-level signal/lower-level trajectory pipelines toward bidirectional mixed control. Vehicle actions should influence regional signal and routing decisions through arrival flow, shock waves, and spillback, while regional controllers should determine where scarce CAV actuation is most valuable. Candidate mechanisms include key-vehicle selection, parameterized continuous actions, hierarchical MPC/MARL, graph-based coordination, and iterative information exchange after network decomposition. The decisive evaluation criterion is whether local interventions reduce network cumulative loss and recovery time, not merely whether they improve one intersection.

### 9.3. Communication-Aware Control and Graceful Degradation

Future controllers should randomize or explicitly model communication delay, synchronization error, packet/feature loss, coverage loss, positioning/perception error, malicious data, computation delay, and actuation refusal during training and testing, then report condition-tagged performance-degradation and recovery curves. Robust optimization, safe reinforcement learning, attack-aware control, trust-weighted fusion, semantic/task-oriented communication, and resilient estimation can enlarge the feasible operating region, but predefined fallback is equally important. A controller that performs well only while V2X is healthy is not resilient; the minimum requirement is safe service retention under degraded information, a documented transition to reduced cooperation, and a controlled path back to coordinated operation when the contract is satisfied again.

### 9.4. A Consistent Disturbance Benchmark for Service Retention, Cumulative Loss, and Recovery Time

Resilience is not a single score. Benchmarks should include crashes/work zones, extreme demand, signal failure, natural hazards, communication/perception failure, cyberattacks, and compound events. Each experiment should specify disturbance intensity, duration, spatial footprint, capacity-restoration trajectory, and clearance process. Online objectives should combine service retention, cumulative loss, and recovery time with efficiency, safety, fairness, and energy, using explicit Pareto trade-offs or stage-dependent weights where necessary.

### 9.5. Adaptive Control Across Penetration Transitions, Scale, and Fairness

Real deployment will involve spatially and temporally varying CAV penetration. Low-penetration stages should emphasize probe value and state estimation; intermediate penetration should target influential vehicles on bottleneck approaches and disturbance-propagation paths; high penetration can support broader cooperative actuation and, where justified, dedicated facilities. Because rerouting, dedicated lanes, and priority control can shift delay to HDVs, public transport, pedestrians, or vulnerable districts, multi-group and spatial fairness should be treated as design constraints rather than after-the-fact indicators.

### 9.6. From Simulation Advantage to Reproducible Engineering Evidence

Future experiments should report training and test networks, demand, driver-behavior parameters, CAV spatial distribution, communication/perception conditions, random seeds, uncertainty intervals, worst-case scenarios, per-decision latency, and computing hardware. When infrastructure-condition sensing is included, studies should additionally report sensor placement, calibration, environmental compensation, uncertainty/validity flags, sampling and communication timing, and how the inferred structural state changes a traffic-control decision. The validation chain should progress from real-data calibration through digital twins/co-simulation, SIL, HIL, and controlled pilots or field trials [23,25,78,79,141]. Table 14 and Figure 12 make the claim boundary at each stage explicit. It is essential to distinguish a platform that operates in the field from a resilience mechanism validated under repeatable disturbances. The latter requires service retention, worst-case safety margin, graceful degradation, cumulative loss, and recovery time under abnormal demand, communication interruption, and actuator or infrastructure failure. The resulting priority research agenda and minimum validation targets are summarized in Table 15.

### 9.7. Limitations of the Review

This review has seven principal limitations. First, it is a structured critical review rather than a registered systematic review; the 2026 WoS/ScienceDirect/IEEE update is auditable, but early database-by-database Scopus/TRID logs cannot be reconstructed fully, so exhaustive coverage is not claimed. The original review workflow did not employ dual independent screening or a prospectively defined inter-reviewer agreement protocol; consequently, formal Cohen’s *κ* statistics are not reported. Borderline records were resolved through author discussion, but some judgment remains unavoidable in a structured critical review. Similarly, the E0–E4 ladder is used as a qualitative interpretation framework rather than an exhaustive numerical classification of all records, and the CiteSpace modularity/silhouette values are descriptive network diagnostics without null-model significance testing. Second, simulation platforms, demand, penetration, disturbance design, baselines, and metrics vary substantially, making reported percentage improvements non-comparable. Third, much of the evidence remains E0–E2 despite increasing real-data, testbed, HIL, and engineering-platform work; evidence on E3–E4 urban-network resilience remains scarce. Fourth, the E0–E4 scale and the control-quality contract are author-defined synthesis tools and have not been externally standardized. Fifth, the 268-record candidate list contains sufficient bibliographic information for year, venue, and author-frequency summaries but not complete affiliation or citation metadata; consequently, country/institution impact, co-authorship, bibliographic coupling, and citation-network analyses were not reconstructed. Sixth, V2X safety, semantic communication, highway-platoon, and generic cyber/validation studies are used only as bridging evidence when their communication or evaluation mechanisms are transferable; they should not be interpreted as direct proof of urban-network recovery effects. Seventh, the 12 self-sensing UHPC papers form a targeted cross-domain bridge set rather than a systematic material-sensing corpus. They support the plausibility of an additional physical-infrastructure sensing layer but do not demonstrate that UHPC-derived electrical signals improve closed-loop CAV traffic recovery, so this translation remains a research hypothesis requiring structural-scale and traffic-control validation.

## 10. Conclusions

The reviewed evidence indicates that CAV penetration should be interpreted as potential rather than automatically realized control authority. The strongest evidence currently concerns mixed-platoon stabilization, vehicle-level disturbance attenuation, and intersection/corridor efficiency. Network-level resilience additionally depends on where controllable vehicles are located, what traffic and infrastructure states remain observable, how HDVs respond, whether communication and computation support the intended cooperation mode, and whether local actions are coordinated with signal, routing, perimeter, and residual-capacity control.

The literature is considerably less mature for closed-loop network recovery under simultaneous physical and information-layer disturbances. Self-sensing UHPC illustrates a possible additional infrastructure-state sensing layer, but the reviewed material studies do not yet demonstrate a complete material-signal–traffic-control–network-recovery loop. It is therefore treated as a prospective observability interface rather than established evidence of resilience improvement.

Accordingly, the effective-controllability indicators, communication-aware control-quality contract, and E0–E4 evidence ladder proposed in this review should be interpreted as author-defined synthesis tools rather than externally validated standards. Future work should test these concepts using explicit disturbance definitions, communication-degradation conditions, service-retention and recovery metrics, computational reporting, and progressively stronger real-data, SIL/HIL, and field evidence.

## Figures and Tables

**Table 1:** Positioning of this review relative to recent CAV mixed-traffic, control, and resilience reviews.

Review	Mixed-Flow Dynamics	Signal Control	Vehicle/Platoon	Joint Control	Disruption/Recovery	V2X/Cybersecurity	Engineering Validation	Organizing Focus
[16] Pan et al. 2024	●	△	●	△	△	△	△	Mixed-traffic effects
[17] Li et al. 2023	○	●	●	●	△	△	△	Urban mixed-traffic control
[18] Matin & Dia 2023	●	△	△	△	△	△	○	Safety and efficiency
[19] Yue et al. 2023	●	○	○	△	△	●	△	Positive/negative impacts
[20] Nisyrios & Gkiotsalitis 2025	○	○	●	○	△	△	△	Optimization-based traffic/fleet management
[25] Ortega et al. 2026	○	○	○	△	●	●	○	4Rs resilience
[21] Majstorovic et al. 2023	○	●	○	○	△	△	△	Mixed-traffic signal control
[22] Zhang et al. 2024	○	●	●	●	△	△	△	RL intersection/joint control
[23] Wang et al. 2026	○	●	○	○	△	○	●	Multi-intersection platform/implementation
[24] Zhang et al. 2026	△	△	○	○	○	●	○	V2X cooperative safety intelligence/SPD framework
This review	●	●	●	●	●	●	●	Local controllability → network recovery

Note: ● = core topic; ○ = systematically discussed; △ = mentioned, but not a principal organizing theme. The symbols are qualitative and indicate scope rather than study quality or effect size.

**Table 2:** Relationship between the historical evidence pool, the 2026 auditable search update, and the final synthesis corpus.

Stage	Records	Role in the Review
Earlier evidence pool	202	Previously screened relevant evidence
2026 auditable update	1601 raw → 1476 unique	Search/audit pool used to identify additional evidence
Incremental relevant records from the update	66	Additional records contributing new relevant evidence
Final synthesis corpus	268	202 earlier + 66 incremental
Evidence tiers	170/89/9	Core/bridging/background–methodological

**Table 3:** Inclusion and exclusion criteria.

Dimension	Inclusion Criteria	Exclusion Criteria
Study context	Urban roads/intersections/corridors/networks; CAV-HDV mixed traffic or directly related resilience control	Single-vehicle perception/planning without traffic-operation implications; highway-only perception algorithms
Control/mechanism	Signal, trajectory, platoon, lane, route, perimeter/network control; disturbance propagation or recovery	Functional development unrelated to urban traffic operations
Disturbance/information	Physical capacity loss, demand shocks, signal/infrastructure failure, communication/perception degradation, attacks	Normal-operation marginal topics that provide no mechanism evidence for RQ1–RQ4
Publication type	Peer-reviewed journal/conference papers prioritized; selected highly relevant frontier material when necessary	Non-auditable secondary material, non-academic web pages, duplicate versions
Interpretability	Traffic context, control object, principal metric, or validation mode identifiable	Insufficient information to judge the study and its claim boundary
Evidence tier	Core: direct CAV/HDV urban-road control/resilience; bridging: transferable disturbance/communication/recovery/validation mechanisms; background: general algorithms/systems	Interpreting non-CAV or non-urban evidence as direct proof of a CAV network effect

**Table 4:** Operational evidence-coding dictionary used for cross-study synthesis.

Dimension	Operational Coding Fields	Why It Matters for Resilience Interpretation
Traffic environment	CAV penetration; HDV/CAV composition; spatial distribution; platoon organization	Separates nominal penetration from the actual availability of controllable vehicles
Information structure	Local sensing; V2V/V2I/V2X; coverage; delay; packet loss; positioning/perception error; trust	Determines observability and functional control authority
Control object	Signal; vehicle trajectory; platoon; lane; route; perimeter; regional capacity	Locates where action is applied and which traffic states can be modified
Control method	Rule/model-based; optimization/MPC; RL/MARL; data-driven; hybrid	Links algorithm class to constraints, scalability, and fallback requirements
Disturbance type	Physical capacity loss; abnormal demand; infrastructure/signal failure; communication/perception failure; cyberattack; compound event	Distinguishes robustness tests from explicit disruption/recovery evidence
Objective	Delay/throughput; safety; energy/emissions; fairness; service retention; cumulative loss; recovery time	Separates nominal efficiency from resilience-oriented objectives
Spatial scale	Vehicle/platoon; intersection; corridor; region/network	Prevents extrapolation from local gains to network recovery
Validation maturity	E0 theory/simulation; E1 real-data calibration/replay; E2 SIL; E3 HIL; E4 controlled field/pilot/FOT	Makes external-validity and deployment gaps visible

**Table 5:** Bibliometric themes visible in the CiteSpace co-occurrence and burst analyses and their role in the cross-scale synthesis.

Theme/Evolutionary Signal	Representative Visible Keywords	Cross-Scale Interpretation	Implication for This Review
Vehicle-level control foundation	Car-following model; adaptive cruise control; connected/automated vehicles	Microscopic actuation and traffic-wave stabilization	Establishes local controllability but does not by itself prove network resilience
Distributed and cooperative control	Decentralized control; cooperative control; string stability; CACC	Coordination expands the influence range of scarce controllable vehicles	Motivates spatial availability and platoon-organization analysis
Signal-trajectory coordination	Trajectory design; traffic signal; trajectory planning; management	Right-of-way allocation and arrival shaping become coupled	Supports intersection-to-corridor joint-control synthesis
Learning and adaptive decision making	Q-learning; reinforcement learning; adaptive dynamic programming	Model-free/adaptive methods broaden high-dimensional control options	Requires stronger safety, generalization, and computation evidence
Communication-aware mixed traffic	Communication delay; communication systems; vehicular ad hoc networks; risk assessment	Information quality becomes part of effective controllability	Motivates cyber–physical co-design and graceful degradation
Network resilience, stability, and validation frontier	Road network resilience; evaluation; real-time systems; stability criterion/analysis; numerical stability; human-driven vehicles	Research focus is moving toward stability-aware, real-time, disruption/recovery, and deployment realism	Reinforces the need for unified recovery metrics, real-time feasibility checks, and higher-maturity evidence

**Table 6:** Comparison of urban road-network resilience quantification approaches.

Method Family	Core Metric/Application	Main Strength	Main Limitation
Topological connectivity	Degree, betweenness, connectivity, redundant paths	Simple and useful for structural vulnerability screening	Does not represent dynamic traffic flow or queue propagation
Traffic-flow performance	Speed, delay, travel time, throughput	Directly reflects user service	Requires an explicit baseline and disturbance window
Resilience triangle/performance curve	Impact depth, cumulative loss, recovery time	Captures both magnitude and duration of loss	Sensitive to baseline and recovery threshold
MFD	Network accumulation, production, mean speed	Low-dimensional network state for perimeter control	Sensitive to heterogeneity and partition quality
Cyber–physical coupling	Road network + communication/perception network	Appropriate for CAV, V2X, and attack scenarios	Cross-layer parameters and validation are difficult to obtain

**Table 7:** Representative intersection- and corridor-scale signal–vehicle coordination methods and evidence boundaries.

Method Family	Control Objects	Main State/Information	Actions/Objectives	Evidence Boundary	Refs.
Coupled optimization/MPC	Signals + CAV trajectories	Position, speed, predicted arrival	Phase/green + acceleration; delay/energy	MPC, Benders decomposition, and back-pressure/discretized trajectory control improve interpretability/decomposability; low-penetration operational evidence emerging, resilience validation limited	[73,74,76,77]
Stochastic/robust hierarchy	Signals + CAV trajectories	HDV trajectory uncertainty	Chance-constrained signal + robust MPC	Explicit uncertainty modeling; mainly simulation evidence	[80]
CoTV-type MARL	Multi-intersection signals + selected CAVs	Local traffic and limited vehicle states	Discrete signal + continuous/parameterized vehicle actions	Key-vehicle control compresses action dimension; safety/generalization remain concerns	[83]
Joint DRL/hierarchical PPO	Signals + CAV/HDV mixed traffic	Traffic/vehicle states, HDV estimates	Discrete signal + continuous vehicle actions; delay/energy/comfort	Low-penetration adaptation, game/asynchronous MARL, and vehicle–road–cloud actor–critic broaden joint control; recovery validation still limited	[84,85,88,89,91,92]
Infrastructure-assisted cooperative driving	Roadside sensing + vehicles	Roadside sensing, V2X, local conflicts	Cooperative driving/intersection management	Depends on sensing coverage and V2X reliability	[93]
Two-level hierarchical optimization	Signals + CAV trajectories + HDVs	Vehicle/platoon states, desired arrivals	PMILP signal + distributed MPC trajectories	Reduces computation; abnormal/resilience validation limited	[75]
Model-guided RL	Signals + CAV/HDV mixed traffic	Traffic states, model knowledge, vehicle states	Model-guided RL joint actions	Model knowledge improves learning; recovery and communication-degradation validation needed	[86]
Corridor/arterial multi-level coordination	Multiple signals + CAV speed/trajectory	Corridor states, signal coordination, CAV states	Bi-level/hierarchical MARL; travel time/energy/safety	Evidence now extends beyond isolated intersections; disturbance-recovery validation limited	[90,94,95]
Signal-lane-trajectory coordination	Signals + lane use + CAV/connected HDV trajectories	Demand, conflicts, platoon/sorting states	Signal timing + lane allocation + platoon/trajectory	Broader spatiotemporal resource coordination; currently dominated by isolated-intersection simulation	[87]

Note: reported percentage improvements are not compared across studies; the table emphasizes control objects, information conditions, and evidence boundaries.

**Table 8:** Engineering bottlenecks, current evidence, and design implications for network-level resilience control.

Engineering Bottleneck	Mechanism	Current Evidence	Remaining Limitation	Design Implication
Local-network coupling	Vehicle control shapes arrivals/waves but cannot allocate network capacity alone	Joint control, vehicle–road–cloud coordination, hierarchical signal-trajectory control, signal-rerouting, capacity allocation, perimeter control [83,89,90,94,95,106,109,110,111,112,134,135]	More network mechanisms are available, but explicit recovery objectives, route/perimeter resources, and communication degradation rarely form one loop	Hierarchical signal–vehicle–route closed loop
Objective mismatch	Normal-operation delay optimum ≠ disruption-loss/recovery optimum	MFD, resilience-oriented signal/perimeter control, incident management, admission-signal recovery tests, dynamic capacity allocation [33,68,69,116,117,120,134,135]	Recovery tests are increasing, but most CAV joint control still optimizes efficiency/energy/stability	Embed service retention + cumulative loss + recovery time online
Communication uncertainty	Delay/packet loss reduce observability and cooperation	Communication-constrained models, communication-aware mixed-platoon control, attack monitoring/fallback, resilient perimeter control, experimental data [49,51,122,128,129,136,137,138,139]	Graceful degradation evidence is stronger, but end-to-end validation against urban-network recovery is limited	Communication-aware control and graceful degradation
Scalability	State/action/communication dimensions grow with network size	Large-scale distributed RL, multi-scale decomposition, perimeter control [110,111,112,140]	Hybrid discrete-continuous actions and real-time cross-scale tests remain limited	Topology decomposition + communication budgets + key-vehicle selection
Penetration transition	Low CAV availability limits the number of actuators	Low-penetration probes, low-V2I robust signals, low-CAV signal-trajectory control, controllability theory, operational data [43,56,70,77]	Spatial availability and actuator location are still weakly evidenced at network scale	Penetration/spatial-availability adaptive hierarchy
Sim-to-real	Behavior, demand, communication, and equipment failures create domain gaps	Real-data calibration, field experiments, scaled physical tests, connected-bus operations, engineering platforms, X-in-the-loop [23,45,46,56,77,79,141]	Urban-network HIL/FOT resilience evidence under repeatable disturbance remains scarce	Digital twin → SIL → HIL → controlled pilot/field

**Table 9:** Communication-aware control-quality contract and minimum stress-test requirements.

Dimension	Observable/Metric	Control Implication	Minimum Stress Test/Reporting
Freshness and synchronization	End-to-end latency; age of information; timestamp skew	Stale or misaligned state can invalidate arrival, platoon, or phase decisions	Sweep delay and clock skew; report the controller threshold at which cooperation is reduced
Reliability and loss	Packet/feature delivery ratio; burst-loss duration	Missing predecessor/RSU information reduces observability and feedback authority	Test random and burst loss; report performance-degradation and recovery curves
Bandwidth and load	Message rate; bitrate; channel occupancy or communication budget	Limited resources require prioritization of control-relevant state	Hold communication budget fixed when comparing compression/selection methods
Coverage and topology	V2V/V2I reachability; connected-neighbor set; RSU blind zones	Changes spatial availability of controllable/observable agents	Inject coverage dropouts and topology changes; record functional CAV availability
Trust and integrity	Confidence/provenance; anomaly/attack flag; false-alarm rate	Corrupted state or commands can generate unsafe coordinated action	Evaluate attack plus false-positive fallback; report safe isolation behavior
Position/perception quality	Localization error; state-estimation uncertainty; alignment error	Affects queue estimation, feasibility margins, and conflict prediction	Stress noise/bias jointly with communication impairment rather than independently
Computation timing	Controller inference/optimization time; edge/cloud delay	Late decisions miss the control horizon even when messages arrive	Report per-decision latency, hardware, deadline misses, and fallback behavior

**Table 10:** Targeted bridge evidence on self-sensing UHPC and its relevance to infrastructure-state observability.

Ref.	Material/Conductive Strategy	Primary Sensing Context	Representative Reported Evidence	Translation Boundary for This Review
[142]	State-of-the-art self-sensing UHPC review	Compression, tension, bending; applications	Synthesizes percolation/electromechanical mechanisms and identifies scale-up, reliability, DAQ/transmission, and structural-integration gaps.	Framework evidence only; not a direct traffic-control study.
[143]	nCB-UHPC	Compressive strain sensing	At appropriate nCB content (about 1.0–1.5%), compressive strength > 140 MPa, slump spread ≥ 170 mm, gauge factor >130.	High material sensitivity; field drift and structural-scale calibration remain necessary.
[144]	Graphene + CNT multiphase/multiscale fillers	Compression; electrical percolation	Percolation strongly changes FCR behavior; reported gauge factors 11–28 while maintaining UHPC-level strength.	Shows threshold dependence; formulation-specific response cannot be generalized directly.
[145]	Graphene + PE-fiber UHPC	Tension/compression; bridge-placement concept	Graphene reduced resistivity and increased self-sensing response; bridge finite-element stress field used to propose sensing locations.	Closest bridge-oriented concept in the set, but no closed-loop field traffic-control validation.
[146]	Hybrid carbon black + carbon nanofibers	Monotonic/cyclic compression	Percolation begins at about 0.5% CB; CNF bridges CB pathways and improves sensing stability/noise behavior.	Useful for stable embedded sensing; acquisition and environmental compensation still required.
[147]	Steel-slag aggregate UHPC; CF/MWCNT comparisons	Direct tension/self-sensing	Iron-rich slag improves conductivity; reported compressive/tensile strengths of about 207/14 MPa and sensing comparable to the MWCNT series.	Potential dual sustainability/sensing benefit; structure-level signal interpretation remains open.
[148]	Recycled carbon fibers	Compression/flexure cyclic piezoresistivity	Resistivity about 1–2 ohm m; gauge factor up to about 38; mechanical gains reported versus no-fiber UHPC.	Promising recycled sensing reinforcement; sensitivity depends on fiber type/content/orientation.
[149]	Cementless UHPC with EAF-slag aggregate	Direct tension/crack sensing	Meets reported UHPC strengths (about 150 MPa compression, 8 MPa tension) and shows low-noise initial-crack sensing, even without carbon fiber.	Sustainable route; authors call for structural-scale health-monitoring validation.
[150]	Hybrid steel fibers + steel wires	Uniaxial tension/crack-width sensing	Tensile strength up to 9.19 MPa; hybrid networks reduce resistivity and support stress/strain/crack correlations.	Relevant to crack-localized monitoring; correlations need environmental and geometric transfer testing.
[151]	Stainless-steel wires + steel fibers with self-heating curing	Flexure; self-heating/self-sensing	Self-heating curing markedly increases FCR/sensitivity; multifunctional SHM and deicing potential demonstrated at specimen level.	Multifunctional benefit, but curing/thermal history becomes part of sensor calibration.
[152]	nCB-UHPC under moisture and sea-salt exposure	Compression/durability of sensing	Moisture reduces strain sensitivity; saline exposure modifies response of saturated specimens and mechanical condition.	Environmental state must accompany the electrical signal in field interpretation.
[153]	Nano-, micro-, and macro-scale carbon black	Monotonic/cyclic compression	Scale-dependent packing and percolation; nCB threshold about 0.5 wt% and more redundant conductive paths; larger CB less effective.	Supports multi-scale design, but reinforces that one calibration cannot be transferred across formulations.

**Table 11:** Translation requirements from self-sensing infrastructure signals to resilient traffic-control states.

Material/Structural Observable	Derived State for Traffic System	Potential Control Use	Required Safeguards/Metadata	Minimum Validation Step
Resistance/resistivity/FCR trend	Strain or stress proxy with confidence bound	Update capacity prior or structural load state	Temperature/moisture compensation; electrode health; polarization handling	Calibrated component test under combined environmental and mechanical loading
Abrupt/irreversible electrical change	Crack initiation or damage-state flag	Trigger lane restriction, inspection, speed reduction, or conservative capacity	False-alarm/false-negative rate; crack localization; hysteresis	Structural member test with independent crack measurement
Distributed multi-channel response	Damage location/affected structural zone	Localize link or lane capacity loss in digital twin	Channel synchronization; sensor ID/location; spatial observability	Bridge/deck/joint demonstrator with controlled damage
Sensing + self-heating state	Thermal/deicing condition and structural response	Winter capacity/safety update and maintenance coordination	Power state; thermal gradients; thermal–mechanical calibration	Environmental chamber + outdoor pilot
Sensor validity/uncertainty flag	Trust level of infrastructure-state estimate	Weight or reject measurement in state fusion; invoke fallback	Drift, aging, communication loss, DAQ faults, maintenance status	Fault-injection test at edge/digital-twin level
Fused structural + traffic state	Decision-relevant capacity and disturbance persistence	Routing, signal/perimeter control, CAV speed/trajectory constraints	Time alignment; causal threshold; operator override; safety envelope	Closed-loop co-simulation/HIL, followed by controlled field test

**Table 12:** Validation-infrastructure matrix for resilient mixed-traffic cooperative control.

Validation Stage	What It Can Establish	Controllable Disturbances/Impairments	Minimum Outputs	Principal Claim Boundary
Analytical/microscopic simulation (E0)	Mechanism, feasibility, sensitivity, upper/lower bounds	Demand, penetration, incident capacity, driver parameters	Traffic trajectory + resilience metrics	External validity and hardware timing remain unknown
Real-data calibration/replay (E1)	Parameter realism and performance on observed states	Recorded demand/trajectories; counterfactual disturbance where justified	Calibration error + replay performance	Limited control over rare or compound disturbances
Traffic-communication co-simulation/digital twin (E0–E2)	Joint traffic-information degradation and repeatable stress tests	Delay, loss, coverage, pose error, demand, capacity loss, attack injection	Traffic + V2X traces + controller actions + recovery metrics	Model fidelity and synchronization assumptions can dominate results
Software-in-the-loop (E2)	Software interfaces, timing, controller integration	Network impairment, sensor feeds, asynchronous messages	Deadline misses + performance + failure logs	No physical controller/actuator behavior
Hardware-in-the-loop/physical testbed (E3)	Real controller latency, communication hardware, interface faults	Packet impairment, actuator/signal faults, real-time computation limits	Hardware timing + safety margin + resilience curve	Scale and behavioral realism may remain limited
Controlled pilot/FOT (E4)	Operational feasibility and field response under bounded conditions	Planned abnormal demand, communication interruption, infrastructure fault where safe	Service retention + safety + recovery + operator constraints	Rare severe disturbances and full network repeatability are difficult

**Table 13:** Synthesis, evidence strength, and major boundaries for the four research questions.

RQ	Synthesis	Relative Evidence Strength	Main Evidence Boundary	Engineering Implication
RQ1	Penetration is potential control capacity; spatial distribution, platoon organization, HDV response, and communication quality determine effective controllability.	Moderate-to-strong locally	Joint evidence under low penetration, spatial heterogeneity, and communication degradation is limited.	Distinguish nominal penetration from functional/spatially available CAVs.
RQ2	Vehicle control suppresses waves and shapes arrivals; signals allocate right-of-way; routing/perimeter control allocates residual capacity; cross-scale coupling is central to recovery.	Strong at intersection/corridor; moderate at network; moderate-to-weak for explicit recovery	Network signal-trajectory, admission/capacity allocation, and incident evidence are increasing, but a unified loop with vehicle actuation, routing/perimeter, communication degradation, and recovery metrics is still missing.	Emphasize mechanisms converting local gains into network recovery.
RQ3	Resilience should move from post hoc assessment into online objectives that report service retention, cumulative loss, recovery time, and absolute service.	Moderate	Some signal/perimeter/incident/recovery studies exist, but most CAV joint control does not jointly optimize the three resilience quantities.	Report P0, disturbance window, threshold η, persistence window Δ.
RQ4	Communication degradation, computation scalability, safe fallback, and sim-to-real transfer are major deployment boundaries.	Moderate-to-weak for high-maturity network evidence	Low-V2I control, delay, attack monitoring, physical testbeds, and operational data are increasing, but urban-network E3–E4 and compound-disturbance evidence remains scarce.	Treat E0–E4 as an author-defined evidence scale, not certification.

**Table 14:** Minimum reporting checklist proposed for future CAV resilience-control studies.

Category	Minimum Reporting Item	Purpose
Traffic composition	Overall CAV penetration, spatial distribution, HDV model/parameters, key-vehicle selection rule	Separate nominal penetration from effective controllability
Disturbance definition	Type, location, intensity, duration, capacity-restoration trajectory, compound-disturbance relation	Make resilience scenarios reproducible
Information conditions	Observation coverage, V2X delay/packet loss, positioning/perception error, attack model	Identify communication dependence and degradation boundary
Baselines and objectives	Baseline control, efficiency/safety/fairness/energy objectives, online resilience objective	Avoid unfair baselines and objective mismatch
Resilience metrics	P0, service retention, cumulative loss, recovery threshold η, persistence window Δ, recovery time	Align physical meaning across studies
Statistics and computation	Random seeds, mean and variance/CI, worst case, per-step latency, hardware	Assess stability and real-time feasibility
Validation maturity	E0-E4 level, real-data source, SIL/HIL/FOT design, cross-network tests	Separate algorithm performance from engineering evidence

**Table 15:** Priority research agenda and minimum validation targets.

Priority Gap	Testable Research Question	Minimum Benchmark	Evidence Target
Spatial controllability	Which CAV locations and information topologies maximize disturbance attenuation at a fixed penetration?	Multiple spatial CAV distributions; bottleneck/crash disturbance; matched nominal penetration	Cross-network simulation + controlled physical or field validation
Signal–vehicle–route closed loop	Do local vehicle actions reduce network cumulative loss when regional routing/signals are co-optimized?	Multi-region network; queue spillback; route choice; signal and vehicle actions	Independent-network tests; real-time computation reporting
Communication-aware resilience	How much service is retained as delay, packet loss, coverage, or trust deteriorates?	Communication degradation curves + physical disturbance; fallback controller	SIL/HIL with reproducible V2X impairment and recovery
Penetration transition and fairness	When should control shift from probe-based estimation to targeted actuation and dedicated facilities?	Low/medium/high penetration with spatial heterogeneity and multiple user groups	Sensitivity analysis + fairness/service constraints
Simulation-to-real transfer	Which policies preserve rank and safety when driver behavior, demand, sensors, and communication differ from training?	Independent networks, domain randomization, hardware latency, abnormal demand	E3 HIL followed by E4 controlled pilot/FOT
Recovery benchmark standardization	Can service retention, cumulative loss, and recovery time be reported consistently across control paradigms?	Shared disturbance intensity/duration/restoration definitions and absolute service baselines	Multi-method benchmark with open scenario/data definitions

## Data Availability

No new data were created or generated in this study. The bibliographic records and information analyzed in this review were obtained from the literature and databases described in the manuscript. Additional information supporting the review methodology and evidence synthesis is available from the corresponding author upon reasonable request.
